# Copper Oxide-Based Photocatalysts and Photocathodes: Fundamentals and Recent Advances

**DOI:** 10.3390/molecules26237271

**Published:** 2021-11-30

**Authors:** Tomasz Baran, Alberto Visibile, Michael Busch, Xiufang He, Szymon Wojtyla, Sandra Rondinini, Alessandro Minguzzi, Alberto Vertova

**Affiliations:** 1 SajTom Light Future, Wężerów 37/1, 32-090 Wężerów, Poland; tommaso.baran@gmail.com (T.B.); szwojtyla@gmail.com (S.W.); 2 Department of Chemistry and Chemical Engineering, Chalmers University of Technology, Kemivägen 10, 41296 Gothenburg, Sweden; visibile@chalmers.se; 3 Department of Chemistry and Material Science, School of Chemical Engineering, Aalto University, Kemistintie 1, 02150 Espoo, Finland; michael.busch@aalto.fi; 4 Dipartimento di Chimica, Università degli Studi di Milano, Via Golgi 19, 20133 Milano, Italy; xiufang.he@unimi.it (X.H.); sandra.rondinini@unimi.it (S.R.); alberto.vertova@unimi.it (A.V.)

**Keywords:** CuO, Cu_2_O, photocatalysis, photoelectrochemistry, water splitting, CO_2_ reduction reaction, hydrogen evolution reaction

## Abstract

This work aims at reviewing the most impactful results obtained on the development of Cu-based photocathodes. The need of a sustainable exploitation of renewable energy sources and the parallel request of reducing pollutant emissions in airborne streams and in waters call for new technologies based on the use of efficient, abundant, low-toxicity and low-cost materials. Photoelectrochemical devices that adopts abundant element-based photoelectrodes might respond to these requests being an enabling technology for the direct use of sunlight to the production of energy fuels form water electrolysis (H_2_) and CO_2_ reduction (to alcohols, light hydrocarbons), as well as for the degradation of pollutants. This review analyses the physical chemical properties of Cu_2_O (and CuO) and the possible strategies to tune them (doping, lattice strain). Combining Cu with other elements in multinary oxides or in composite photoelectrodes is also discussed in detail. Finally, a short overview on the possible applications of these materials is presented.

## 1. Introduction

**Figure 1:**
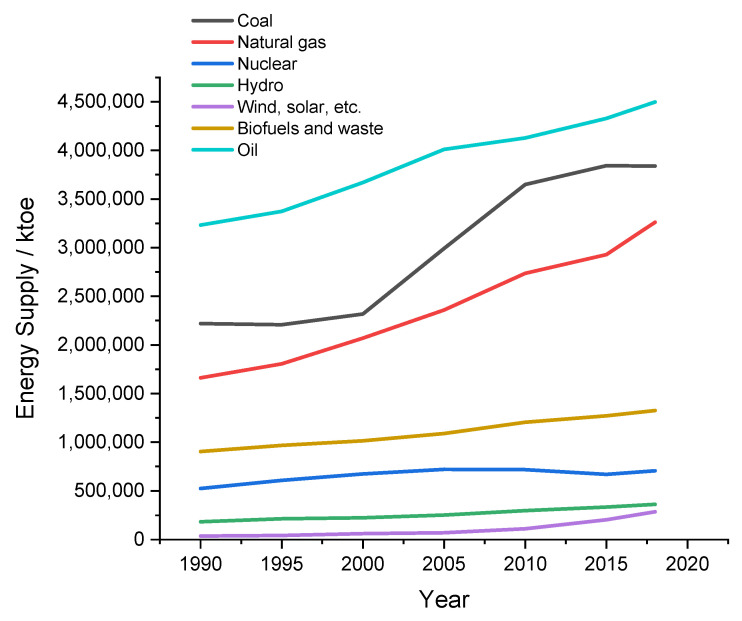
Energy supply source share in the last 30 years. Based on International Energy Agency, IAE, data from IEA (2021) [Energy Supply by Source], https://www.iea.org/data-and-statistics (accessed on 16 September 2021), All rights reserved; as modified by the Authors of the present paper.

Energy production is clearly a key requirement in the progress of technologies, well-being and more generally for sustainable human activities. According to the International Energy Agency (but as is evident to everyone), Figure 1, the main source of electricity derives from the combustion of fossil fuels.

The growth of the human population and the progressive increase of energy consumption in all continents are increasing the request of energy, that has exponentially grown in the last two centuries. This put in evidence the main limits of a system based on fossil fuels, that are limited on our planet and whose combustion is at the bases of major air pollution and of the green-house effect mostly due to CO_2_ intense emissions. The predicted effects of the latter are dramatic, as evidenced by the report of the Working Group I of the sixth assessment report by the Intergovernmental Panel on Climate Change (IPCC) [1], that states “The likely range of total human-caused global surface temperature increase from 1850–1900 to 2010–2019 is 0.8 °C to 1.3 °C, with a best estimate of 1.07 °C”.

More frequent hot extremes, intensified water cycles (incl. rainfalls and floods), permafrost and glaciers thawing, loss of seasonal snow cover and extreme sea-level increases (due to both ice melting and thermal expansion from ocean warming), are just some of the consequences and are likely to get worst. To give an example, the IPCC regional atlas predictions (https://interactive-atlas.ipcc.ch/, accessed on 16 September 2021) indicate that, in the medium term (2041–2060), the Mediterranean Sea level will rise to about 0.3 m, that will increase to 0.7 before 2100. The same report evidenced that the temperature growth is not homogeneous on the planet, reaching the highest value at the arctic ocean. If mitigation measurements are not applied, the Earth surface temperature will increase globally by about 4–5 °C in 2080–2100.

This motivates several states and international organizations to promote actions aimed at mitigating or reversing this trend. In 2015, United Nations promoted the Paris Agreement, a legal binding treaty signed by 196 parties to reduces CO_2_ emissions and limit the temperature well below 2.0 °C (possibly 1.5 °C) over the pre-industrial level. Among the signatories, European Community countries agreed to set a long-term strategy to be climate-neutral (zero CO_2_ emissions) by 2050. An intermediate target will consist of cutting greenhouse gas emissions by at least 55% by 2030.

Shifting towards a renewable energy-based economy is therefore central. It has been calculated that the amount of energy that can be collected by day (sun)light is more than sufficient to satisfy the humans’ needs [2], but the call for efficient ways of exploiting renewable energy sources requires the development of new technologies in several fields, including chemistry.

Indeed, harvesting sunlight for converting it into other forms of energy (thermal, electric) is a key challenge. Typical limits of renewable sources are their spatial limitation and their temporal oscillations. This requires that the energy is stored to be used elsewhere or when the source is not available.

Among the different ways to reach this goal, converting sunlight into a fuel is one of the most promising thanks to the efficient transportation and storage of chemicals. In this sense, the conversion of existing infrastructures (e.g., those used for oil) represents an additional advantage.

Mimicking nature in converting sunlight and simple reactants (e.g., water) in an endergonic reaction to produce a fuel has been termed artificial photosynthesis. These are the reasons why the development of artificial photosynthetic systems for converting solar energy and water into solar fuels such as molecular hydrogen (H_2_) has attracted and still attracts enormous interest to lead the energy economy to a higher sustainability. H_2_ presents high energy density when compressed to its liquid state. Carbon dioxide reduction to methane, formic acid or methanol is another example of solar fuel production; however, its technology readiness level is lower in comparison with the production of hydrogen fuel. The use of semiconductors in the photo-electro-chemical (PEC) water splitting is one of the most promising approaches in terms of scale-up technology to yield highly pure H_2_. In PEC water splitting, in the simpler configuration, a semiconductor immersed in solution and coupled to a counter-electrode is illuminated by solar light. Light absorption by the semiconductor causes the formation of electron/hole pairs. The two photogenerated charge carriers are separated and can drive two half-reactions thanks to the electrical field generated within the semiconductor at the semiconductor/liquid junction (SCLJ). Quite often, this requires the help of an external applied potential (bias), unless a tandem (or “Z”) system composed by an n-type and a p-type semiconductors are used in the same cell.

For n-type semiconductors, the anodic reaction (that proceeds thanks to the transfer of holes to the electrolyte) occurs at the semiconductor’s surface, while the cathodic one is driven at the counter-electrode. In a symmetric fashion, a p-type semiconductor can work as a photocathode, where the cathodic reaction (transfer of electrons to the electrolyte, i.e., water reduction to hydrogen) occurs while the anodic one occurs at the counter-electrode.

In this review we will focus our attention on the use of copper-based semiconductors as photoelectrodes. We will firstly demonstrate why Cu oxides deserve attention (Section 2 and Section 3) and we will review synthetic methods for preparing these materials, also considering all possible modification (doping, addition of under/overlayers or cocatalysts) to increase the performance of the final photoelectrode. Finally, (Section 4) we will describe all the possible applications in which these materials have been tested so far.

## 2. Copper Oxide Based Materials

As mentioned, photoelectrochemical water splitting is one of the most promising routes for renewable hydrogen generation, being a one-step process for sunlight-to-H_2_ transformation in mild conditions [3,4,5,6].

In photoelectrochemical water splitting (PEC-WS), the oxygen evolution reaction (OER) represents the anodic reaction:

(1) $$H_{2}O\;\overset{\;}{\to }\frac{1}{2}O_{2}+2H^{+}+2e^{-}\;$$

While the hydrogen evolution reaction (HER) is the cathodic one:

(2) $$2H_{2}O+2e^{-}\;\overset{\;}{\to }{\;H}_{2}+2{\mathrm{OH}}^{-}\;$$

An efficient semiconductor should present the following features:

- A sufficient sunlight absorption for high yield generation of excited states inside the semiconductor.
- A suitable band gap energy (*E*_g_) to enable sunlight absorption.
- An efficient charge separation to avoid recombination and ensuring a high quantum efficiency.
- Proper bands position with respect to the equilibrium potentials of the desired half-reactions.
- Show high stability and photostability.

Semiconductors able to perform reactions (1) and (2) without undergoing photodegradation typically have a wide band gap that limits the absorbed portion of the solar spectrum (e.g., TiO_2_ with a 3.2 eV band gap can absorb only in the UV range) [7].

In the research of suitable photocathodes, Cu_2_O is one of the most studied ones since:

- It presents a 2.17 eV band gap [8]. This value is low enough to have the proper energy to drive water electrolysis by visible light absorption.
- It presents suitable bands position, allowing both the HER and the OER [9] (see below).
- It is made of abundant and low-cost elements.
- It is non-toxic, allowing for easier industrialization. This is an advantage if compared to other semiconductors for PEC-WS containing As, Cd and other toxic metals.
- It can be easily and reproducibly synthetized by several methods, including electrodeposition.

As anticipated, the bands position in Cu_2_O satisfies the above mentioned requirements, having a conduction band (CB) edge potential, *E*_c_, of about −1.16 V, far above the energy corresponding to the H^+^/H_2_ couple (−0.65 V) and *E*_v_ (about +1.0 V) slightly higher than the water oxidation potential (+0.82 V) at pH = 7 [10,11].

Although Cu_2_O is the most promising Cu-based semiconductor, the corresponding Cu(II) oxide, cupric oxide—CuO, often co-exists with Cu_2_O being co-synthetized during the preparation of Cu-based photoelectrodes. Most of this manuscript will deal with the preparation, the properties tuning and the activity of Cu_2_O-based materials. However, it is worth spending a paragraph on CuO as well.

CuO physicochemical properties and its activity towards PEC water splitting have been very recently discussed in a dedicated review [12]. However, it is worth to summarize the main properties of CuO, because of its relevance in the topic discussed in the present work and for the frequent co-existence of Cu_2_O and CuO in promising photocathodes.

Like Cu_2_O, CuO is a p-type semiconductor (due to Cu vacancies), its structure is monoclinic, space group C2/c [13], and absorbs visible light thanks to a bang gap of about 1.7 eV [14]. CuO is more conductive than Cu_2_O [15]. However, n-CuO can be also synthetized [16].

CuO is a promising photoactive material, particularly for the degradation of organic pollutants, while it possesses synergistic effects when coupled with Cu_2_O for CO_2_ reduction reaction and for water splitting. In particular, CuO has been often proposed as an overlayer for Cu_2_O to promote electron transport towards the electrolyte, thus reducing the probability of charge recombination and increasing the lifetime. When used as a photocathode, this combination lead to impressive photocurrents up to −19.12 mA cm^−2^ at −1 V versus RHE [17]. Cu_2_O/CuO systems have an extended absorption spectra compared to pure Cu_2_O: While the latter have an absorption edge at about 600 nm, the former have a stronger absorption in the visible region—up to near-infrared (NIR), whose edge is at about 900 nm, due to the low band gap energy of CuO [18].

The activity of this system can be enhanced by partially reducing it by hydrogenation, leading to the formation of a thin layer of Cu(OH)_2_ [19], or by deposition of a carbon-based film to reduce charge recombination and promote charge transport [20].

Interestingly, a CuO/Cu_2_O composite can be formed starting from pure CuO and partially reducing it under operative conditions. This material retains a good photocurrent for 6 h at 0.35 V (RHE) [21].

A similar procedure carried out without the FTO (fluorine doped tin oxide coated glass) support, lead to a CuI/CuO core/shell powder that show both photocathodic and photoanodic properties with high faradaic efficiencies in the first case [22]. Interestingly, thanks to the different pH dependence of the band edges of the two component, the electronic features of this material become pH-tunable [23].

In the following, other examples of the use of photoactive CuO will be revealed.

### 2.1. Cu_2_O

Cu_2_O crystals have the so-called cuprite structure, a cubic Bravais lattice with the symmetry of the 224th space group (*O*4h, Pn3m). This structure is limited to few other compounds such as Cd(CN)_2_, Ag_2_O, Zn(CN)_2_, and Pb_2_O. Inside the unit cell the oxygen ions are located on a bcc sub-lattice, while copper ions on a fcc sub-lattice. Inside the cell, the copper ions are on the vertices of an oxygen tetrahedron and they are two-fold coordinated with the oxygen ions (*D*_3*d*_ site symmetry), whereas the oxygen ions are fourfold coordinated with copper (*T_d_* site symmetry) [24,25].

Two interpenetrating sub-lattices compose the cell lattice in which each oxygen is tetrahedrally surrounded by four copper atoms and each copper is directly connected with 2 oxygen atoms in a linear configuration [26]. The spontaneous formation of Cu vacancies inside the lattice that creates holes in the valence band (VB) is the source of conduction.

**Figure 2:**
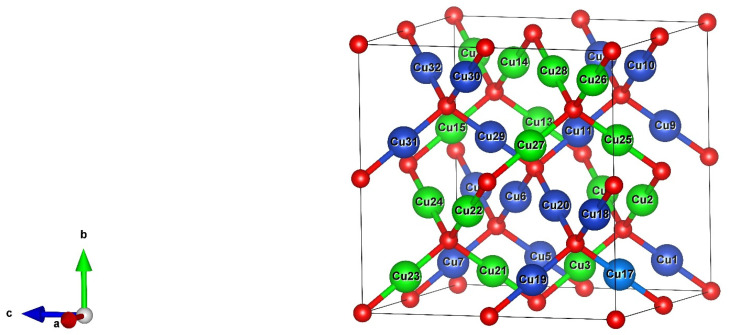
VESTA representation of a Cu_32_O_16_ supercell. Oxygen atoms are in red while the Cu atoms of the Table 1. Provides the lattice constant and the crystal structure. A transformation of Cu_2_O from the cuprite to a hexagonal structure occurs at the high pressure of 10 GPa (*a* = 4.18 Å). This hexagonal structure changes in the pressure range from 13 to 18 GPa into another hexagonal one, with CdCl_2_ type structure. Up to 24 GPa, the highest pressure studied, no decomposition of Cu_2_O into Cu and CuO was observed. Anyway, the only structure here considered is the one stable at atmospheric pressure.

Figure 2 shows the 2 × 2 × 2 supercell with the two interpenetrating sub-lattices (one in blue and one in green).

It is important to note that the reported values are almost temperature independent, as Cu_2_O is characterized by a very small expansion coefficient (changes in the lattice constants are less than 0.5% from 0 to 600 K). However, Cu_2_O is characterized by a negative thermal expansion below 300 K [27]. In Table 2 are listed the main Cu_2_O parameters.

**Table 1:** Tabulated lattice parameters for Cu_2_O obtained by XRD. Data from [28], with the permission from the American Physical Society.

Lattice Parameters	Values	Units
Lattice constant “a”	4.27	Å
Cu-O bond length	1.85	Å
O-O bond length	3.68	Å
Cu-Cu bond length	3.02	Å

**Figure 3:**
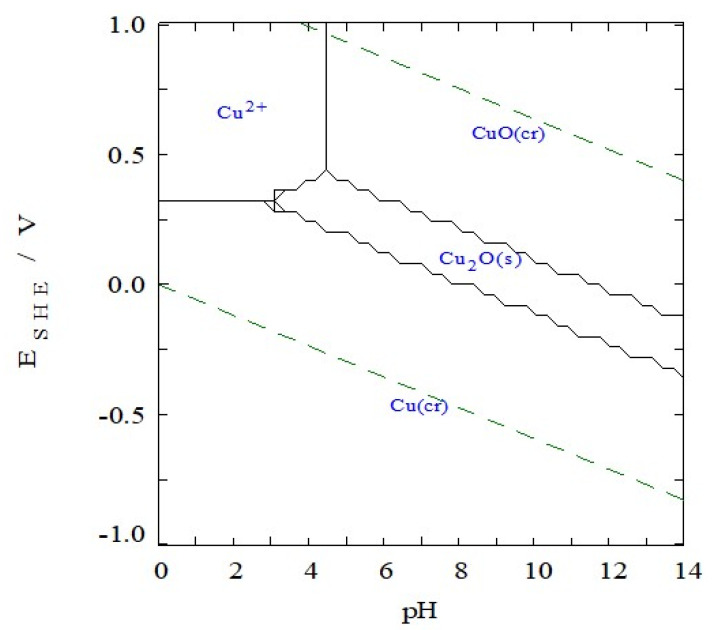
Poubarix diagram of Cu for Cu^2+^ 70 mM at 25 °C from Hydra-Medusa^®^ software (version V.1), where (s) stands for “solid” and (cr) for “crystalline”.

Figure 3 shows a Pourbarix diagram of Cu calculated using the Medusa^®^ software (https://www.kth.se/che/medusa/, accessed on 16 September 2021). Here is possible to identify the thermodynamic range of stability (in terms of pH and potential) for the different species. From the diagram it is possible to notice the narrow region of stability of the Cu_2_O, while the CuO oxide is much more stable from pH above 5. The water stability range is, as usual, represented between the dotted lines at 0 and 1.23 V at pH 0. The concentration of Cu^2+^ here considered was the one present in the electrodeposition bath of Cu_2_O, discussed later as one of the most promising preparation routes.

**Figure 4:**
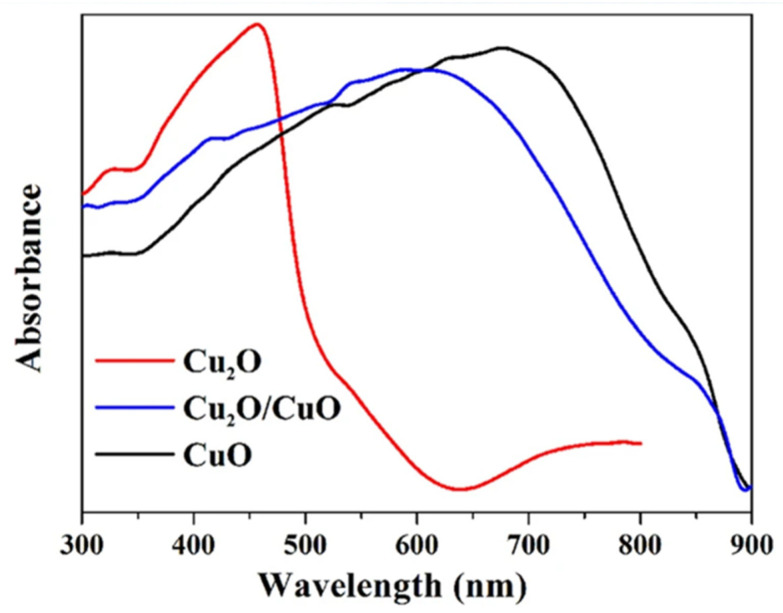
UV–vis diffuse reflectance spectra of the pure Cu_2_O (read line), pure CuO (black line), and Cu_2_O/CuO (blue line) composite films prepared on FTO substrates. Reprinted from [18], with the permission of Springer Nature.

The optical properties of a material are of extreme importance in photo-electrochemistry and thus PEC-WS applications. Cu_2_O has a direct band gap of 2.17 eV [8]. However, according to the absorption spectrum (Figure 4 [18]), it starts to only absorb the light above approximately 2.4−2.5 eV (500—520 nm). This energy is related to the dipole allowed transition between the higher valence band and the second lower CB [30]. Figure 4 also shows the absorption spectra of CuO and of a Cu_2_O/CuO system: the former having a wider absorption range thanks to the lower BG, the latter combining the optical properties of the two oxides.

Cu_2_O band gap was considered as related to d10-d10 interactions between the Cu 3d states of adjacent Cu ions [31,32,33]. Recent studies showed that these interactions do not exist [34]. Instead, thanks to a combined analysis of the density overlap region indicators (DORIs), crystal overlap Hamilton population (COHP) and the density of states (DOS), it was demonstrated that the chemical bonding in Cu_2_O is mainly characterized by covalent bonding. Within this configuration the electrons are delocalized over Cu-O-Cu moieties through two-electron and three-center bonds [34]. Following this analysis, both the CB and VB edges are determined by covalent σ* antibonding bonds between Cu 3d^4^sp and O 2sp^3^ states.

**Figure 5:**
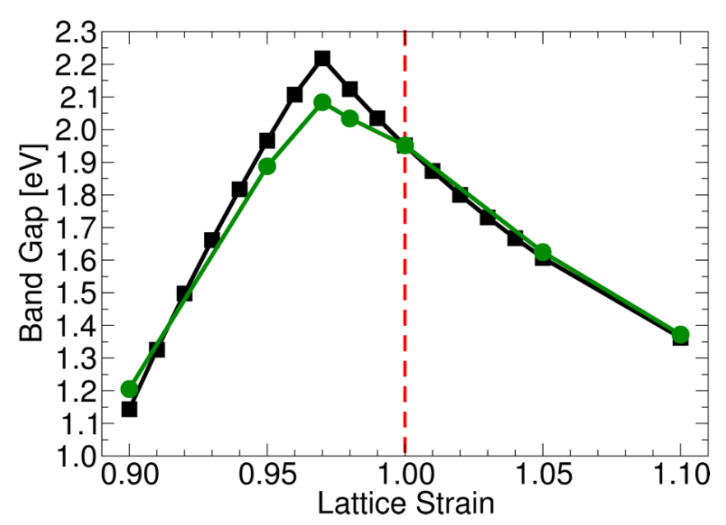
Summary of the influence of 2D (green circles) and 3D (black squares) strain on the band gap. Reprinted from [34] with the permission of the American Chemical Society.

The delocalized nature of the multicenter bond determining the VB and CB edges can be exploited to manipulate the band gap both through inducing strain onto the Cu_2_O lattice [34], and by doping with inert same-valent ions in high concentrations [35]. Both effects have been observed in quantum chemical modeling studies. Imposing, for example, tensile strain onto the lattice of Cu_2_O results in a weakening of the Cu-O bond and thus in a reduced energy gap between bonding, non-bonding and anti-bonding states. This in turn translates into a reduced band gap (Figure 5). The opposite effect is observed when compressing cuprite by up to −3%. increasing the tensile strain further, Cu_2_O starts to show an anomalous behavior, in the sense that the band gap starts to decrease again. This was associated with the presence of delocalized Cu 4sp, which starts to dominate the CB edge.

**Figure 6:**
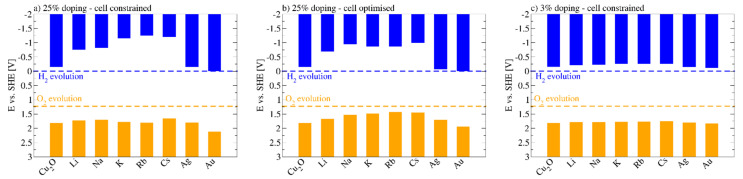
The band gaps of pure and alkali metal doped Cu_2_O: (**a**) 25% doping at unrelaxed Cu_2_O unit cell; (**b**) 25% doping in relaxed unit cell; (**c**) 3% doping in unrelaxed unit cell. Band alignments are stated as electrochemical potentials (E) versus the standard hydrogen electrode (SHE). Reprinted from [35] with the permission of Elsevier.

Doping Cu_2_O with high concentrations of inert group I metals, such as Li or Na, on the other hand, disrupts the delocalized two-electron and three-center bonding network and effectively localizes the electrons in a confined space. This in turn converts into an increased band gap (Figure 6) [35].

Decreasing the concentration, thus increasing the space in which the electrons are delocalized, reverts this effect. Similarly, doping with Ag and Au ions does not affect the band gap to a significant amount. This is not surprising when considering that these ions can contribute to the two-electron and three-center bonding network.

#### 2.1.1. Preparation Methods

Different preparation methods are reported in literature to produce a valid Cu_2_O layer for photo-application. The most valid ones are reported below.

Thermal oxidation of metals is a widely used method for the synthesis of high-quality oxides. In this case, the procedure involves the oxidation of a high purity copper foil from a few minutes up to several hours depending on the required final thickness of the Cu_2_O layer. Temperatures are in the range between 1000–1500 °C under pure oxygen atmosphere or mixed gas atmosphere (like Ar + O_2_) [36]. The obtained Cu_2_O is polycrystalline with different grain structures according to the chosen experimental conditions. During the thermal process two reactions can occur:

(3) $$4\mathrm{Cu}+O_{2}\overset{\;}{\to }2{\mathrm{Cu}}_{2}O\;$$

(4) $$2{\mathrm{Cu}}_{2}O+O_{2}\overset{\;}{\to }4\mathrm{CuO}\;$$

The formation of a mixture of two major oxides, CuO and Cu_2_O, is always possible and thus the partial pressure of oxygen during the annealing process must be strictly controlled. The formation of Cu_2_O occurs first while longer oxidation time are needed for CuO to appear [37]. An alternative method starts from a Cu foil that is converted in Cu(OH)_2_ by the use of 0.125 M (NH_4_)_2_S_2_O_8_ followed by thermal reduction. Nanocorals of Cu_2_O [38] or nanosized Cu_2_O are obtained if the oxidation is performed in KOH and a dehydration step is performed [39].

One of the most attractive methods for large scale and high-quality production of Cu_2_O is electrodeposition. The advantages of this method are that it is cheap, can easily work on different substrates and allows the tuning of the material properties and morphology working with parameters like: The applied potential, the current, the temperature, and the pH of the bath [40]. The first electrochemical synthesis of Cu_2_O was presented by Stareck [41]. Successively, many other authors developed different synthetic procedures using different copper precursors electrolytes and electrochemical parameters [42,43,44].

Mao et al. used a solution of 0.01 M Cu(NO_3_)_2_ + 0.1 M NH_4_NO_3_ with a current density of 0.5 mAcm^−2^ for 60 min at 313 K. The resulting photocurrent was quite low [45]. Wan et al., using 0.02 M Cu(Ac)_2_ and 0.1 M CH_3_COONa aqueous solution with 1.5 mM KCl, were able to control the shape of the grains with pH modulation, but the conductivity of the so obtained material was n-type [40]. Zhao et al. performed electrochemical oxidation of a Cu foil in a solution of 280 gL^−1^ NaCl and 0.1 g L^−1^ Na_2_Cr_2_O_7_, with pH adjusted between 8 and 12 by 1.0 M NaOH [46]. They show a net decrease in the band gap value probably as a result in the particles morphology [46].

To the best of our knowledge, the highest results in terms of photocurrent were obtained using a CuSO_4_ solution with lactic acid and the pH shifted to basic value (usually 12) [47,48,49,50]. This is a development of the recipe derived from Golden et al. [48,49,50,51]. The photocurrent obtained with this recipe largely changes according to the specific publication. For example, Nian et al. [52] obtained a maximum photocurrent of −0.025 mAcm^−2^ on FTO, the Graetzel group [48,49] obtained values as high as 2 mAcm^−1^, while Visibile et al. reached 1.6 mAcm^−1^ tuning the properties of the metallic underlayer below the semiconductor [53]. This highlights the extreme importance of controlling every parameter in the deposition with high accuracy.

PEC-WS’s performances of systems fabricated with this method are nowadays one of the most efficient and leading to the highest photocurrents. This method guarantees that the semiconductor layer is very homogenous and has highly tunable properties. For example, it is possible to control the oxide morphology and the particle’s size [54,55] by simply varying selected bath conditions like potential, temperature and pH [54,55]. It is also important to cite the controlled oxidation of a copper foil in different solutions [56,57]. The result obtained with this method in terms of photocurrent falls behind the one previously described.

Other synthetic procedures includes reduction of copper-amine complex solution with glucose under microwaves irradiation [58], the use of different surfactants [59,60,61,62,63,64] and micelles [65] mostly to control the morphology of the particles. Using these procedure, Cu_2_O nanowires and nanocrystals [66] with cubic [67,68,69], cuboctahedral, truncated octahedral, octahedral [70], and multipod structures [71] have been prepared [72]. Surfactant free synthesis have also been developed to reduce interferences from these surfactants [73,74,75]. Solvothermal [76,77] and sol-gel [78] methods [76,77] have also been tested. Wet chemical routes [79,80], thermal evaporation [81,82], chemical vapor deposition [83], sonochemical synthesis [84], hydrothermal [85,86,87] and electroless [88] methods are only few of the other alternatives methods for the synthesis of this semiconductor. Another interesting technique for the preparation of thin films is sputtering. This preparation route allow high homogeneity, low cost and easy synthesis [89]. With this synthesis there is usually no available data about the produced photocurrents.

#### 2.1.2. Cu_2_O Advantages and Disadvantages for PEC-WS System

In a PEC-WS system, electrochemically deposited Cu_2_O is a preferential choice because:

- Cu_2_O has a 2.17 eV band gap. The value is high enough to have the proper energy for hydrogen evolution, but not so high, thus the material can absorb in the visible range of light. Compared to material like TiO_2_, able to absorb only in the UV due to their large BG (~3 eV), this is a great advantage.
- Cu_2_O has a proper bands position for both HER and oxygen evolution reaction OER. In most scenarios the material is used as a photocathode.
- Cu_2_O is a low-cost semiconductor that originate from abundant precursors. This allow a sustainable scale-up of the electrode material production.
- Cu_2_O is non-toxic. Compared to other semiconductors for PEC-WS containing As, Cd and other toxic metals, this is of great interest from the environmental point of view.
- Electrochemical synthesis allows wide control over different parameters, being able to obtain high-performance electrodes.
- Electrodeposition is a cheap and fast method for the preparation of a large amount of electrodes.

**Figure 7:**
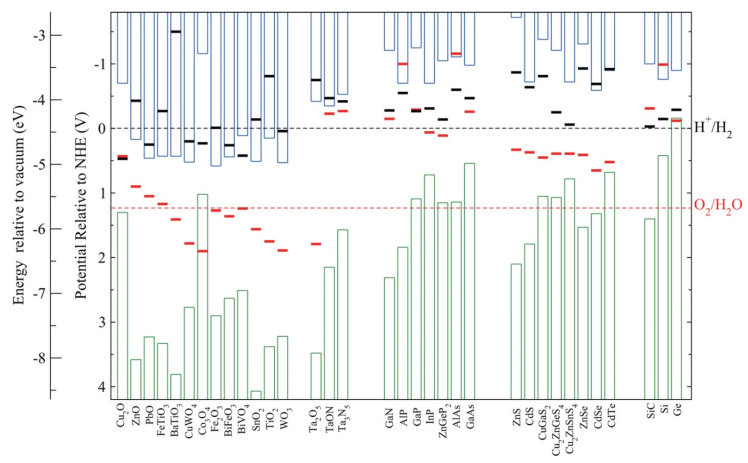
Comparison of band gap, band energies and redox potentials for different semiconductors for PEC-WS. Reprinted from [9] with the permission of the American Chemical Society.

Looking in detail at the Cu_2_O band position in Figure 7, we can see that is one of the few materials with a narrow band gap still able to achieve both the HER and the OER (CB more negative than H_2_/H_2_O redox potential and VB more positive than O_2_/H_2_O redox potential).

From the same picture, the main disadvantage of this material is also evident. The redox potentials for material reduction/oxidation lie inside the band gap. Thus Cu_2_O can undergo the photodegradation process, where, in this case, both holes and electrons can interact with the material provoking, respectively, oxidation and/or reduction.

To extend material lifetime towards photodegradation and enhance the material’s performances, two different approaches have been used in the literature: doping and protection with an overlayer.

Creation of a protective heterojunction is instead a way of protecting Cu_2_O from the photodegradation mechanism. The idea behind is to use an overlayer able to quickly remove electrons from Cu_2_O thanks to the redox cascade principle before they react with the material itself.

#### 2.1.3. Stability of the Semiconductor

Photodegradation is the worst of the many undesired processes occurring after electron-hole couple creation because it reduces the material activity with time. This problem is widely common in semiconductors for PEC-WS.

**Figure 8:**
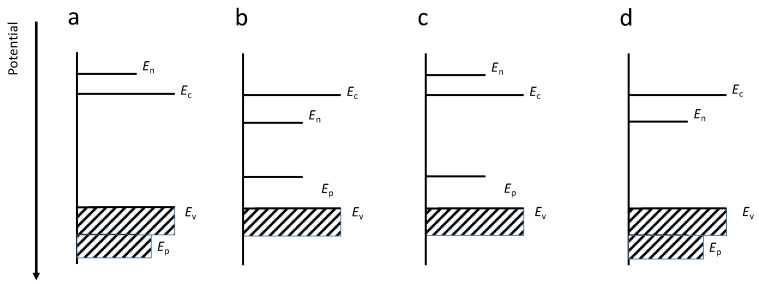
Models of thermodynamic stability. (**a**) Thermodynamic stability, (**b**) possible anodic and cathodic photodegradations, (**c**) possible anodic degradation, (**d**) possible cathodic degradation. *E*_v_—valence band, *E*_p_—redox potentials of anodic decomposition reactions, *E*_n_—redox potentials of anodic and cathodic decomposition reactions, *E*_c_—conduction band.

There are four different levels of stability for a semiconductor according to the VB and CB levels, with respect to the redox potentials of the material (Figure 8):

(a) Thermodynamic stability: The redox potentials of anodic and cathodic decomposition reactions (*E*_p,_ *E*_n_ respectively) are more positive (less negative) and more negative (less positive) than the VB and CB edges, respectively.

(b) Anodic and cathodic degradations: Both redox potentials lie inside the BG. The material can be degraded by electrons reduction and holes oxidation.

(c) Anodic degradation: The CB edge potential is more positive (less negative) than that redox potential so the semiconductor is stable from cathodic degradation. Anodic corrosion by holes still affect the material by self-oxidation.

(d) Cathodic degradation: Holes do not affect the material but the high energy electrons excited in the CB can perform undesired material reduction.

The photodegradation is in competition with the desired processes of surface electrons transfer. The favored reaction is the one with less energy required but also kinetics plays an important role and thus it is not always easy to predict the final behavior of a material [9]. Other important parameters influencing the stability are the chosen electrolyte and its concentration and pH, temperature, impurity levels and other setup parameters (e.g., stirring that can affect the rate of electrode processes).

#### 2.1.4. Vacancies Formation

The Cu^+^ ion external electronic structure is 3*d*^10^*4s*^0^, with the *4s* orbitals only a little higher in energy than the *3d* levels. The Cu *3d* levels form the VB of Cu_2_O and the empty Cu *4s* levels form the CB [90,91]. This is different from most metal oxides, which have O *2p* states at the top of the valence band. From DOS analysis, it was demonstrated that Cu_2_O has a direct gap at the center of the Brillouin zone (Γ point) [92].

**Figure 9:**
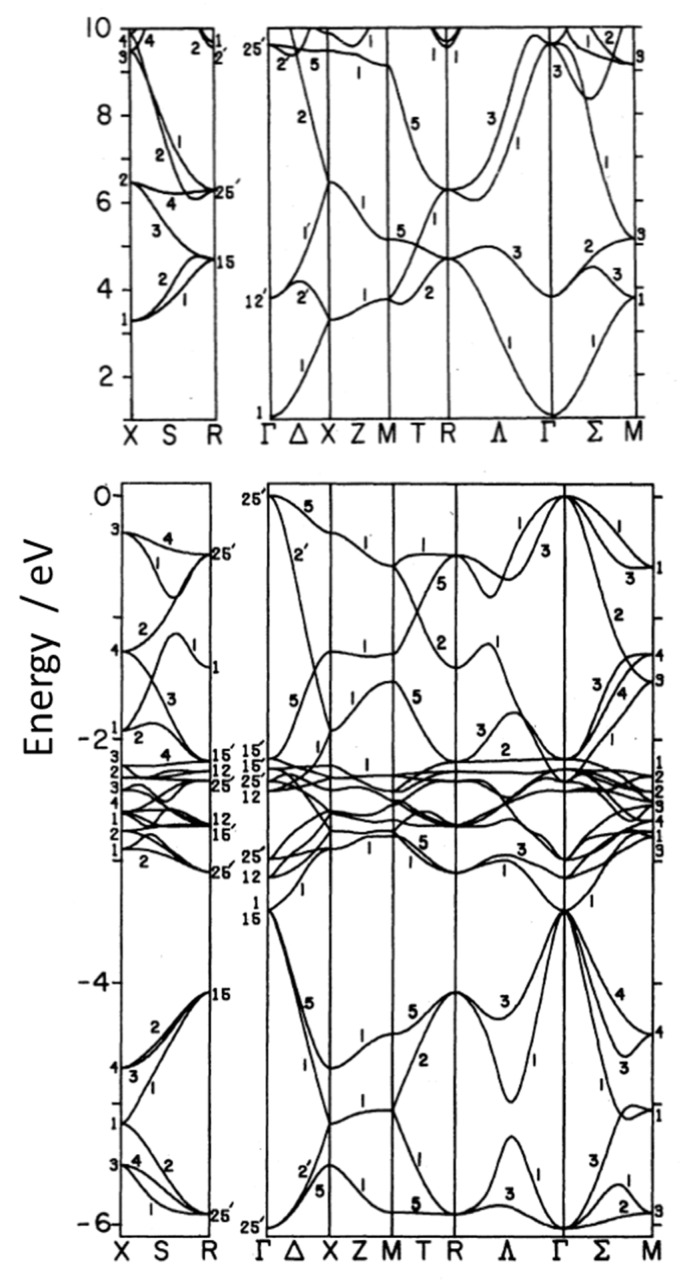
Cu_2_O band structure plot calculated with the LDA self-consistent method. On the top, conduction band; on the bottom, valence band. Reprinted from [93] with the permission of the American Physical Society.

**Figure 10:**
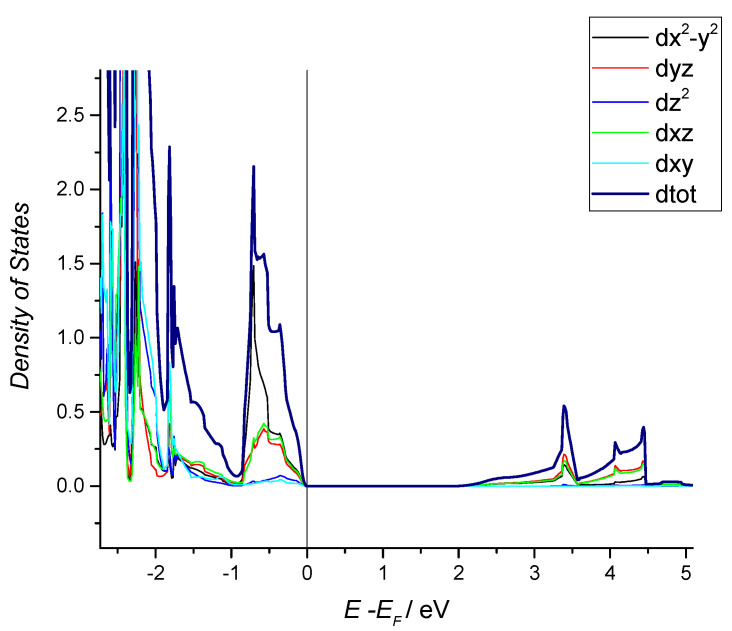
Split density of states (DOSs) of Cu_2_O obtained with VASP and HSE06 method. All the energies are reported to the Fermi level one.

Kleinmann et al., in their DOS analysis Figure 9 [93], have the typical underestimated BG of LDA methods (Figure 9 and Figure 10). Figure 10 instead shows the BG calculated with hybrid functional, a result much closer to the real one with the different contribute of d-orbitals in the valence and conduction band. The real measured energy gap is *E*_g_ = 2.1720 eV at 4.2 K, obtained as the limit of the yellow exciton series and it decreases with temperature [93]. Using a range separated hybrid functional (HSE06), we recently found a BG of 1.95 eV [34].

As mentioned earlier, one of the key parameters for PEC-WS materials is their band positions with respect to the water oxidation and reduction potentials. Water reduction and oxidation potentials must fall within the valence band edge (*E*_v_) and conduction band edge (*E*_c_) for the reaction to be thermodynamically favorable. The closer the *E*_c_ energy to the vacuum level, the stronger the reducing power. In the same way, the lower *E*_v_, the higher the oxidizing driving force. The bands alignment of Cu_2_O satisfies all the above mentioned requirements.

Conductivity for Cu_2_O comes from copper vacancies that create acceptor states within the BG at energy values of 0.3−0.5 eV above the top of the VB. Copper vacancies (V_Cu_’s) can reach concentrations up to 10^20^ cm^−3^, but the free holes’ concentration at 25 °C is usually only around 10^18^ cm^−3^ because not all the vacancies are ionized. The formation enthalpies of the defects suggest that many parameters like the O_2_ partial pressure, temperature, and Fermi energy can largely modify their concentration. The annealing temperature can, moreover, increase the minority carrier lifetime up to one order of magnitude [94,95,96].

The formation of a Cu vacancy occurs easily and spontaneously as the computed energy is 0.38 eV [90], a quite low value if compared to similar materials. Once one vacancy is already present, the formation energy for the next one changes according to the reciprocal position of the two vacancies. Nolan et al. computed the different energies to found the most favorable position that is on a different Cu_2_O network (Table 3) [90].

The most stable configuration for dopant and Cu vacancy is the one where the Cu vacancies are clustered (i.e., separated by the internetwork Cu-Cu nearest neighbor distance, with one vacancy on the same Cu_2_O network as the dopant and the second vacancy in the other Cu_2_O network). When the number of vacancies is increased to three, two of the three Cu vacancies are in the same Cu_2_O network. Similar for four Cu vacancies, where three of them are found in the same Cu_2_O network, and so on.

A neutral oxygen vacancy, able to add electrons to the system, could compensate the holes but taking away a single neutral oxygen atom tetrahedrally coordinated to a Cu atom requires 3.08 eV, much more with respect to a neutral copper vacancy. Other combinations of Cu and oxygen vacancies present even higher formation energies. In other words, the Cu vacancies compensation by formation of oxygen vacancies is not favored process. Moreover, another study suggests that hole traps formation is not linked with Cu vacancy, avoiding any negative impact on the conductivity [97].

### 2.2. Doped Copper Oxides

Bulk doping with ions (metal or non-metal) is a simple method of semiconductor photosensitization (shifting the absorption edge towards lower energy light) and improvement of photocatalytic activity. Dopant ions provide an additional energy level (donor or acceptor levels) within the band gap of the semiconductor [98]. Light-induced electron excitation from the valence band to the acceptor level, or from the donor level to the CB, requires a lower photon energy compared with the excitation of bare semiconductor. Moreover, a dopant ion can act also as a charge trap, leading to prolongation of the lifetime of the charge carriers and towards an enhancement of the photocatalytic activity. On the other hand, doping leads also to several negative effects: (i) decrease of carrier mobility owing to the formation of the strongly localized additional states within the band gap, (ii) increase of the rate of photogenerated charges recombination [99,100]. Copper oxides, as semiconductors with a narrow band gap, are active in visible light and, therefore, the purpose of the doping is not associated with sensitization to visible light, as is usually the case of wide band semiconductors (e.g., TiO_2_, ZnS).

Doping is the addition of impurities into the material lattice with the aim of modifying the band gap and the bands position. As a result of this, the fraction of light absorbed by the semiconductor as well as carriers’ number and mobility can be increased. Moreover, doping might include some strain in the material lattice as a result of the different ionic sizes of the dopant. This strain might result in a modified band gap, as suggested by Visibile et al. using DFT calculations [34].

Cu_2_O presents a spontaneous p-type conductivity and it is also a compensated material where both intrinsic acceptors and, in smaller number, donors co-exist. The compensation ratio N_A_/N_D_, (the acceptor concentration over the donor concentration), is usually just slightly larger than 1 and always lower than 10. A similar condition found in other semiconductor has been explained with the self-compensation mechanism [101]. The higher is the number of donor impurities inserted in the material, the more the acceptors formation energy is reduced; in this way, donors are always less than acceptors. The nature of the compensating donor is still controversial (simple candidates could be oxygen vacancies) and also their identification as intrinsic defects is not assessed. Many authors claim n-type doping to be impossible because of the self-compensation mechanism, others claim to be able to obtain an n-type behavior, for example, with Cl-doping [102,103,104,105].

Cation doping changes the material crystal structure of two interpenetrated Cu-O networks kept together by non-bonding Cu-Cu interactions. The Cu_2_O BG can be increased or decreased with the use of the appropriate dopant, because any change is the sum of different mechanisms:

(i) The size of the dopant cation. It affects the Cu-Cu interactions in the Cu_2_O host lattice (e.g., Sn^2+^ increases the Cu-Cu distances because of its larger ionic radius thus increasing the BG by reducing the metal character of the material). In general dopants with ionic radii larger than Cu^+^ (like Ba^2+^, Sn^2+^, Cd^2+^, In^3+^, La^3+^, Ce^4+^ etc.) produce strong structural distortions around the dopant site. Dopants with ionic radii smaller than Cu^+^, such as Al^3+^, Ga^3+^, Ti^4+^, and Cr^4+^, show almost no structural distortions.
(ii) The alignment of the dopant electronic states with those in the VB or CB of Cu_2_O (e.g., dopants like In^3+^ (larger than Cu^+^) or Al^3+^ (smaller than Cu^+^) cause a decrease of the BG because of unoccupied *3s* states with much lower energies than the Cu *4s* orbitals).
(iii) The introduction of dopant ionic states within the gap with the possible formation of an intermediate band (IB).
(iv) Charge localization through insertion of insulating ions such as Li^+^ or Na^+^. This typically results in an increased BG.

The sum of all these effects affects the final material behavior. The isovalent doping of Cu_2_O using Ag^+^ or Au^+^, for example, produces little structural distortions into the Cu_2_O lattice and BG is not affected. The hole mobility is only slightly affected because the *d*^10^ levels of Au^+^ and Ag^+^ are too low in energy to have a strong interaction with the *d*^10^ levels of Cu^+^ [97].

Regarding the structural distortions introduced by cations doping in Cu_2_O, the *d*^10^-*d*^10^ interactions between Cu atoms are of paramount importance in determining the BG and the width of the VB. When the *d*^10^–*d*^10^ interactions are suppressed, *E*_g_ is indeed increased. This results shows that the physical properties are related not only to 2D interactions but also on its global spatial arrangement. Thus Cu-Cu 3D interactions must be improved to reduce the BG [32,106].

Doping with cations larger than Cu^+^ increases the band gap, while maintaining the cubic structure because it distorts the crystallographic lattice and thus decreases 3D Cu-Cu interactions resulting in a BG increase. The distortions induced by the dopant in the Cu_2_O lattice depend obviously on the size of the dopant. In the case of early transition metals (TMs), the TM-Cu bond length (d_N-N_) is shorter than the Cu-Cu bonds in the pure Cu_2_O host. This phenomenon has been noted in several works. The explanation is that the deviation from the *d*^10^ configuration results in stronger TM-Cu bonds [32,33].

Cu^+^ in Cu_2_O behaves as a soft Lewis acid, with metallic Cu-Cu interactions creating high-lying VB states and low-lying CB states, resulting in a higher conductivity and lower transparency with respect to the oxides of harder Lewis acids (e.g., Na_2_O). Thus, as suggested by literature [31], the use of cations from hard Lewis acid tends to increase the band gap, while using cations from soft Lewis acid leads to a reduction. This strongly depends on the purpose for the Cu_2_O preparation. An electrode for PEC-WS should present a quite low BG, instead a p-type semiconductor for transparent conductive oxide (TCO) [107] should possess a quite wide BG. The latter are used as transparent electrodes in flat panel displays and in solar conversion devices. Examples of TCO are indium tin oxide (ITO), ZnO or FTO. For their preparation the synthesis of a semiconductor with large band gap and still high conductivity is mandatory [32]. Nowadays, all TCO are n-type semiconductors, and the development of a hole-conducting material (p-type) like Sr:Cu_2_O [107] could generate new applications for this kind of material, like functional windows able to transmit visible light but at the same time to generate current due to the absorption of UV photons. The BG tunability is, thus, a great advantage of Cu_2_O.

In Cu_2_O, aliovalent dopants compensation is achieved by forming Cu vacancies. For a dopant of oxidation state n+, (n − 1) Cu vacancies are needed to compensate, the subsequent (n-th) Cu vacancy is used to dope the system p-type. Aliovalent cations can thus be used to increase the number of vacancies. The values of vacancy formation energies with different dopant cations are reported in Table 4 with data coming from Nolan et al. [31].

In the column headed $E_{n-1}^{vac}$ is shown the formation energy for the first Cu vacancy compensating for dopant with oxidation state M^n+^; in the column headed $E_{n}^{vac}$ is presented the formation energy for the Cu vacancy that creates the p-type for dopant with oxidation state M^n+^.

H and passivation: Tabuchi et al. [108], with an exposure time of 30 min, pH_2_ = 1 m Torr at a temperature T_sub_ = 300 °C [108], succeeded to reduce the carriers concentration from 1.7 × 10^16^ to 2 × 10^15^ cm^−3^ and to increase the mobility from 5.3 to 28.9 cm^2^/(V s). Thus hydrogen has the ability to passivate acceptors. According to the authors, mobility increases by the lower effect of ionic impurity scattering due to the lower carrier concentration and then by the passivation of the dangling bonds at the grain boundaries. Passivation of non-radiative recombination centers can be also obtained by cyanide treatment with an increased holes density [109].

Alkaline Metals: Alkaline doping mostly ends in reduction of the direct band gap moving the acceptor level edge to the maximum of the VB. Moreover, AFM showed a carrier density and electrical conductivity increase as well as a reduction in the photocurrent with the dopant ion size. Our computations indicate that alkaline metal doping affects the BG through a combination of geometric and electronic effects. The formers are the result of differences in the ion size and effect Cu_2_O in an identical manner than simply straining the oxide whereas the latter are a result of the destruction of the semiconducting network of two-electron three-center bonds spanned by the Cu-O-Cu bonding network. Both effects only notably affect the oxide at very high concentrations [35]. On the other hand, Caballero-Briones et al. claimed that the main effect of the alkaline substitution of copper atoms is the polarization of O states, with a reduction in the insulating gap and splitting of the density of states just below the Fermi level [110]. In particular, with Na the resistivity is decreased from 1.2 × 10^6^ to 330 Ωcm, and the carrier concentration rises from 5.1 × 10^14^ to 1.7 × 10^18^. A small reduction of the BG (from 2.19 to 2.04 eV) due to the creation of an impurity acceptor level above the valence band was recorded [111,112,113].

Chlorine: Results on this material shows a conductivity about one order of magnitude greater than the undoped material. Carriers concentration’s (holes) measurements as a function of temperature proved chlorine to be a donor, substituting the oxygen, and an acceptor as well, sitting in an interstitial position [105].

Nitrogen: It slightly opens the band gap with the formation of an intermediate band (IB) at about 0.9 eV from the VB. The IB is mostly composed by hybridization between the N-*2p* states and the Cu-*3d* states. According to some authors, in this doping, nitrogen atoms substitute oxygen ones behaving as a p-type dopant [114], but other papers are in disagreement, considering it too difficult to substitute O with N because of the larger radius of nitrogen [115]. Anyway all the studies found improvements in the absorption coefficient spectra [116,117] as a result of the presence of an intermediate band (IB).

Zinc and indium: According to different authors, the Zn-doping can reduce the lattice constant of Cu_2_O, giving an increase of the BG when Zn act as a donor impurity [118,119]. Some authors found that Zn-doped Cu_2_O has a larger resistivity than the undoped one [120], while others found an improved carrier concentration and charge transfer, despite an extremely low photocurrent [121]. Heng et al. [122], noticed a shape evolution of the Zn-doped Cu_2_O microcrystals. In particular, increasing the relative concentrations of the Zn precursor leads to a decrease in the numbers of facets. A small increase in the band gap with the increase in Zn concentration was recorded too. Indium acts as a donor impurity in Cu_2_O [120] by increasing the lattice constant and the band gap. In some cases this can lead to the formation of n-doped Cu_2_O [120].

Silicon: Si is usually coordinated with four oxygen atoms, instead of two like copper. Therefore, Si acts probably not as a substitutional impurity but creating SiO_2_ inclusions [123]. This leads to a lowest resistivity of 12 Ω cm and a Hall mobility of about 10 cm^2^/(V s). No information on photocurrents were given.

Silver: Ag-doping generates minimal changes in the band gap and band gap energies. Improved conductivity and photocurrent density (up to four times) with respect to undoped samples were recorded [124]. Ag in Cu_2_O can, owing to its chemical similarity to Cu and the presence of d-orbitals, directly replace Cu in the structure without significantly influencing the bonding situation. Computations indicate that the bonding situation is somewhat more covalent, which allows for a stronger delocalization, thus could explain the experimentally observed higher conductivity and higher charge carrier generation, which in turn provides a greater photo-response [35]. High dopant concentration decreases the performances because of recombination centers that inhibit the increased charge separation efficiency [124].

Iron: Fe-doping shows a reduced experimental BG from 2.5 to 2.2 eV with enhanced photocurrent at minimum dopant concentration (2%). Higher concentrations generate more defects that, as recombination centers, reduce the charge separation. The recorded improved conductivity could be also due to the presence of Fe *3d* states in the doped sample [125,126].

Gold: Doping with gold produces only a minimal change in the optical absorption edge (−1.6% of the undoped value). Gold is also helpful for improving the transmissions of photoelectron and vacancy thanks to the formation of an impurity level, which can enhance the visible absorption, and reduce the absorption efficiency of UV light [127]. Au has been found to reduce recombination phenomenon too. Computations indicate that the electronic structure of Au-doped Cu_2_O is affected in an almost identical manner to what has been observed for Ag [35].

Magnesium: Mg-doped Cu_2_O gives a great boost to the conductivity. The doped thin films with morphology changes showed an electrical resistivity decrease from 202 to 6.6 Ωcm, due to the increase of charge-carrier density. The BG increases a little to 2.4 eV compared to intrinsic cuprous oxide, but, on the other hand, the charge carrier density increases from 4.5 × 10^15^ up to 8.1 × 10^17^ cm^−3^ with increasing Mg content from 0% to 17% due to the enhanced formation of copper vacancies. These two properties combined lead to a general decrease of resistivity [128]. Other authors claimed that the similar ionic radius of Mg with respect to Cu, together with its d^5^ configuration, should make Cu_2_O less prone to form trap states [129].

Strontium: Strontium doping induces a significant improvement of the electrical properties, with a thin film resistivity reduced down to 1.2 Ωcm at 25 °C with a strontium content of 5–7%. However, at higher strontium concentrations, the resistivity increases again even after annealing. The room temperature densities of the free hole lies in the range of 1.2 × 10^15^ to 2.8 × 10^17^ cm^−3^, depending on the Sr content and on the post-deposition annealing [130].

Other authors find that an increase in Sr content decreases resistivity from 10^6^ to 10^2^ Ωcm for film with 5–6% of Sr. Unlike Nolan’s calculation [90], no increase of the band gap was recorded in the experiments while morphology is affected by Sr content indeed [131].

Manganese and Nickel: Mn-doping results is an increased resistivity at room temperature for the doped sample [132]. In another case, the resistivity of the doped samples was lowered by a factor of 2 with respect to the undoped ones [133]. Undesired results were found also for Ni-doped Cu_2_O. When the Ni atoms enter the interstitial sites they produce scattering centers of the neutral impurity, resulting in low mobility. The mobility in Ni-doped films monotonically increased with increasing deposition temperature, while the mobility in undoped films decreased [134]. Thus Ni impurities do not affect the carrier concentration but only the mobility.

Single cationic doping with the generation of acceptor states in the band gap is not the only possibility when talking about Cu_2_O doping. Indeed, these states could behave like recombination centers reducing the efficiency of the photocatalyst. Instead of mono-doping, anionic and cationic co-doping could improve the properties of the material. This is also of help in case of poor dopant solubility. The simultaneous incorporation of p-type and n-type dopants (compensated co-doping) will lower their formation energy [135].

The calculated defect formation energy shows that co-doping is energetically more favorable than mono-doping due to Coulomb interactions and charge compensation effects. Recent results were reported with TiO_2_ [136], ZnS, Bi_2_WO_6_ [137,138] and of course on Cu_2_O [10] with DFT calculations. On Cu_2_O, the best combination found was composed by Sn + B doping, and this leads to a reduction of the forbidden band gap and an increased optical absorption.

**Figure 11:**
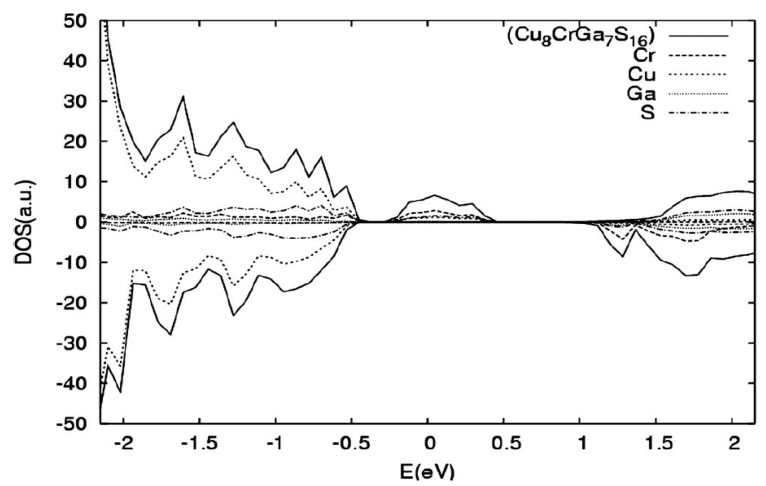
DOS of a chalcopyrite-doped system with intermediate band gap. Figure reprinted from [147] with permission from the American Society for Mechanical Engineering.

Another interesting property of the doped system is the introduction of an intermediate band (IB) inside the BG of the semiconductor because of the energetic level of the dopant. These systems are called intermediate band solar cells (IBSCs) (Figure 11). Firstly discovered by Luque [139,140] in 1997, they are still studied today because of their great advantages [141,142,143]. The IB acts as a steppingstone for electrons coming from VB. In this way, electrons can be excited also by photons, having less energy than the BG but at least the energy between IB-CB and IB-VB. With this trick the total carriers generation increase and the light absorption is expanded significantly [144,145,146].

On Cu_2_O systems Malerba et al. [148] and other authors [119,121,149] obtained interesting results with N-doped Cu_2_O both from computational and experimental points of view.

**Figure 12:**
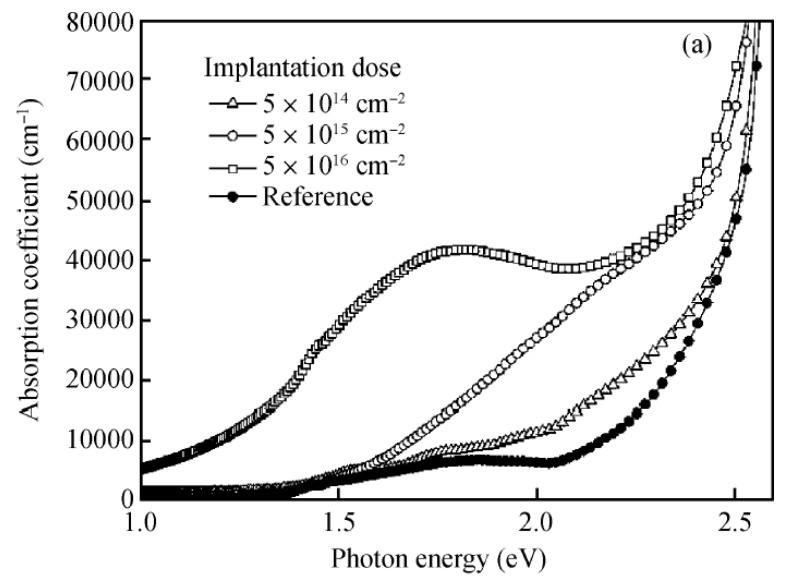
Adsorption spectra of different doses of N-doped Cu_2_O and undoped films obtained from experimental results. Reprinted from [116] with permission from the Institute of Physics (IOP) Publishing.

**Figure 13:**
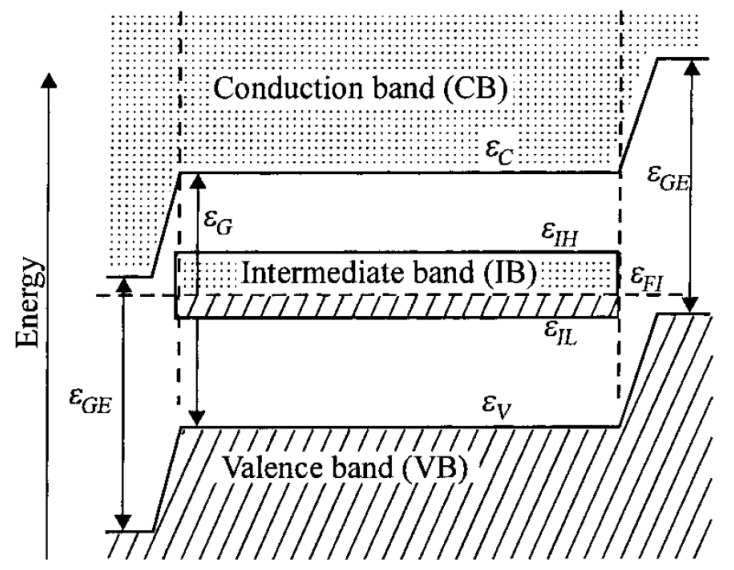
Sketch of an intermediate band (IB) energy band diagram in thermal equilibrium showing the energy band extremes. Dashed and dotted regions correspond to electron-filled and empty bands, respectively (at 0 K). Reprinted from [150] with the permission of John Wiley and Sons.

A new absorption peak below the BG with a red-shift of the absorption band edge is visible in Figure 12 from the paper of Ping et al. [116]. The higher number of useful photons can be used for the electron-hole pair generation and thus improve the performances of the material. A schematic representation of the mechanism is given in Figure 13.

To obtain an efficient IB, some requirements have to be fulfilled [139,150,151,152,153]:

- To have strong light absorption for transition to and from IB, it has to be half-filled and lie on the Fermi level [147].
- Must be clearly separated both from VB and CB to avoid recombination.
- Split the original BG in two sub-band gaps of approximatively the same width to maximize the use of photons.
- Should not be a center for non-radiative recombination.
- It must have small dispersion and must not be a discrete level.

### 2.3. Ternary Copper Oxides

Delafossites are an example of ternary oxides, which have a quasi-two-dimensional layered superlattice structure. In this structure, the linear O–A–O dumbbell layers and oblique BO_6_ octahedra layers are alternately arranged [154]. Delafossite materials based on copper (CuMO_2_; where M = Fe, Rh, Cr, Al, Ga) has attracted great attention as a possible cathodic photomaterials for solar fuel production because of the small band gap energy, high onset potential and good stability in aqueous electrolytes. Crucial electronic properties of copper-based ternary oxides are summarized in Table 5. On the other hand, poor charge transport properties yield in a low efficiency of photoelectrochemical H_2_ evolution [149]. Copper-iron oxide is one of the few oxides that naturally displays p-type conductivity but, unlike Cu_2_O, CuFeO_2_ is stable under reductive conditions [155]. The conduction band of CuFeO_2_, located at around −0.4 V vs RHE, is suitable to reduce water to hydrogen under light. The most commonly used synthesis methodology consists of the solid-state reaction between iron and copper oxides in oxygen-free atmosphere at 800 °C. In order to improve the photoactivity of delafossite material, a unique fabrication strategy has been proposed. The CuFeO_2_ was prepared by hybrid microwave annealing (HMA) as a post-treatment [149]. The HMA method resulted in interstitial oxygen doping into CuFeO_2_ lattice to form CuFeO_2_^+1.5δ^, thereby increasing charge conductivity and improving electron-hole separation (more than four times higher photocurrent density in comparison with conventional thermal annealing). Prévot et al. proposed citrate-nitrate sol-gel method, whereby metal nitrates were mixed with citric acid, ethylene glycol and ethanol [155]. This procedure resulted in thin film obtained directly on FTO (fluorine-doped tin oxide) glass, which revealed high photocurrent densities (up to 1.4 mA cm^−2^), by using sacrificial electron acceptors and an advantageous photocurrent onset at +0.9 V vs RHE. This material absorbs light in a broad wavelength range—the IPCE onset being at λ = 830 nm. Further studies reported high density of surface states, which may cause Fermi level pinning at the SCLJ [156]. These surface states act as electron traps, resulting in an inversion of the depletion layer upon filling, both in the dark and light conditions, thereby promoting charge recombination at the surface.

Other promising p-type copper-based delafossite photoelectrocatalysts are: CuCrO_2_, CuAlO_2_, CuGaO_2_ and CuRhO_2_. The delafossite CuCrO_2_ photocatalyst can be prepared using hydrothermal and solid-state reaction methods resulting in a significantly different materials. A hydrothermal CuCrO_2_ sample is characterized by higher purity, lower crystallinity, smaller particle size and higher photocatalytic activity in comparison with samples prepared by a solid-state method [157]. The CuAlO_2_ photocathodes ware fabricated by electrodeposition of Cu^2+^ and Al^3+^ onto FTO glass, followed by thermal treatment [158]. CuAlO_2_ possesses suitable electronic properties for H_2_ evolution under solar light and exhibits a photocurrent onset potential of +0.9 V vs RHE, along with a faradaic efficiency of ca. 70% at +0.3 V, which can be significantly improved by the addition of sacrificial hole scavengers. A hydrothermal method has been applied also to prepare CuGaO_2_ [154]. The CuGaO_2_ electrode exhibits the potential for hydrogen production, long-term stability and large photocurrent density in PEC tests. The CuGaO_2_ electrode exhibits the potential for hydrogen production with a small overpotential (−0.1 V) and long-term stability. Bocarsly’s group described a CuRhO_2_ material that is a more active photomaterial in oxygenated conditions, due to O_2_-driven self-reparation [159]. Under irradiation, the CuRhO_2_ can undergo reductive deactivation, but in the presence of oxygen this regenerates the active photocatalyst again. CuRhO_2_ enables evolution of H_2_ with ca. 80% Faradaic efficiency.

A literature describes also numerous other ternary oxides characterized by relatively high activity in photocatalytic or photoelectrochemical reactions. Many of these examples are summarized in an recent review [161]. Among them there are copper (I)-based compounds (e.g., CuNbO_3_, Cu_2_Ta_4_O_11_ and Cu_3_VO_4_) and copper (II)-based oxides (e.g., CuAl_2_O_4_, CuCo_2_O_4_, CuFe_2_O_4_, CuMn_2_O_4_, CuV_2_O_6_, Cu_2_V_2_O_7_ and CuWO_4_). Furthermore, it is worth mentioning a few other materials. Berglund at al. reported CuBi_2_O_4_ photocathodes fabricated by a straightforward drop-casting method [160]. It was reported that the optical absorption of CuBi_2_O_4_ is rather weak, as they exhibit a gradual instead of sharp increase in absorption for light energies above the band gap value—this reduces the photocurrent density. Another limiting factor is poor charge carrier (mainly holes) transport, which reduces the obtainable photocurrent density of two orders of magnitude. Copper ferrite, being a p-type semiconductor, was applied as a photocathode in water splitting reaction. CuFe_2_O_4_, prepared directly on FTO glass via the sol–gel method followed thermal treatment, generated a photocurrent density of 1.82 mA cm^−2^ at 0.4 V vs RHE [162]. This result is significantly more encouraging than those of many other copper oxide materials. This has been associated to the reduced oxygen vacancy concentration and thus facilitated charge transport and interfacial charge transfer efficiency.

### 2.4. Quaternary Copper Oxides

Quaternary oxides contain copper and two other metal cations in the oxide structure. Literature shows significantly lower interest in quaternary oxides materials in comparison with ternary oxides. The Cu_2_BiVO_6_ consists of a 4:1:1 combination of Cu_2_O, Bi_2_O_3_ and V_2_O_5_. Cu_2_BiVO_6_ has strong visible light absorption, starting at 600 nm and band gap energy smaller than that of BiVO_4_ due to a mixing of the Cu-3d orbital with the V-3d conduction band of BiVO_4_ and to the mixed Bi-6s [163]. Under illumination, this material generates both anodic and cathodic photocurrents, attributable to water oxidation and reduction, respectively. Zhou et al. reported that another quaternary oxide, CuBiW_2_O_8_, exhibits a strong absorption of visible light and it has a promising values for mobility of photoexcited carriers compared to ternary oxides such as a BiVO_4_ and CuBi_2_O_4_ [164]. CuAlGaO_4_ is a visible-light-active materials for hydrogen photocatalytic evolution from H_2_S with quantum yield of 10.8% at 550 nm [165]. Further increases of quantum efficiency were achieved when CuAlGaO_4_ was loaded with nickel oxide. Double perovskite compounds Sr_2_CuWO_6_ and Ba_2_CuWO_6_ were investigated as photocatalysts and photoelectrocatalysts [166].

### 2.5. Composites of Copper Oxide with Other Semiconductors (Heterostructures)

**Figure 14:**
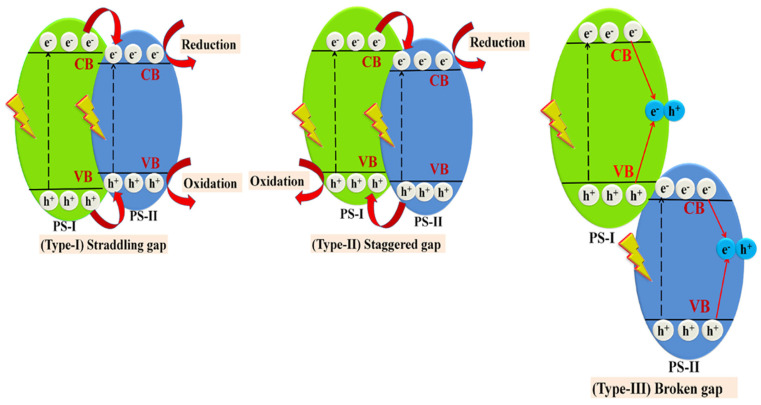
Schematic heterojunction growth of type-I straddling gap, type-II staggered gap and type-III broken gap. Reprinted from [167] with the permission of Elsevier.

Heterojunction occurs between two different semiconductors with suitable band gap alignment leading to the enhanced transfer of photogenerated charge carriers by reducing recombination rates of electron-holes pairs. This approach is considered as the most promising method leading to an improved photoactivity [167]. This becomes particularly important in the case of copper oxide, because of its tendency to photocorrosion. The heterojunction materials can be classified into three different types based on the band structure (Figure 14):

- Type-I (straddling gap)—CB energy in the semiconductor “I” is higher, while VB potential is lower than in the semiconductor “II”, so that both electrons and holes can migrate towards the semiconductor “II”.
- Type-II (staggered gap)—CB and VB energies of the semiconductor “I” are lower than in case of the semiconductor “II”, so electrons can migrate towards the semiconductor “II”, while holes move in the opposite direction, leading to spatial electron-hole separation.
- Type-III (broken gap)—VB energy in the semiconductor “I” is lower than the CB energy in the semiconductor “II”, resulting in no transfer or separation of electron–hole pairs.

Copper oxide/titanium oxide is one of the widest studied photocatalytic composites in various application. TiO_2_/Cu_2_O represents a type-II heterojunction, while TiO_2_/CuO shows a type-I interaction. Copper oxide/titanium oxide composites are typical examples of p-n junction heterostructures. The CuO-TiO_2_ was reported as a photocatalyst of organic compounds degradation [168,169,170], photoelectrocatalyst of water splitting [171,172] and carbon dioxide reduction [173]. Copper and titanium oxides have multiple roles in composite. The most important advantage results from the formation of p-n junction. The n-type material (TiO_2_) has a high electron concentration, while the p-type (CuO) a high hole concentration, thus electrons diffuse towards the p-type region and holes towards the n-type one, leaving behind exposed charges (positive or negative) on dopant atom sites. An electric field between the positive and negative ion cores is called a depletion region and the presence of this effect is facilitating the charge separation between electrons and holes [171]. Furthermore, the presence of copper oxide in titanium material extends the range of light absorption [170], acts as a cocatalyst [169] and can affect the reaction mechanism [173]. General mechanism of photoactivity of the CuO-TiO_2_ composite is based on the transfer of photoinduced electrons from the conduction band of TiO_2_ to the conduction band of CuO. A different mechanism occurs in the case of Cu_2_O-TiO_2_. Since the CB potential of Cu_2_O is more negative than the one of TiO_2_, under irradiation electrons from CB of Cu_2_O are transferred towards TiO_2_ [174]. Similar to the case of CuO-TiO_2_, Cu_2_O-TiO_2_ exhibits an improved activity of photo(electro)catalytic reaction in comparison with bare oxides. Recent literature examples include: Cu_2_O/TiO_2_ nanotubes or Cu_2_O/TiO_2_ octahedron for H_2_ evolution under sunlight [175,176], Cu_2_O/TiO_2_ or Cu–Cu_2_O/TiO_2_ heterojunction photocatalysts for dyes degradation [177,178].

The CuO/Fe_3_O_4_ type-II heterojunction was studied towards the photocatalytic degradation of organics [179]. An enhanced photocatalytic activity of the heterojunction in comparison with bare CuO and Fe_2_O_3_ was assigned to the improved separation between charge carriers, a declined band gap energy, a large surface area, and the suppressed recombination of charge carriers. The studied heterojunction showed extended durability upon working conditions up to five times.

Copper-zinc oxide nanoflowers prepared by hydrothermal route showed the hydrogen evolution in pure water, while in the presence of sacrificial hole scavenger (triethanolamine) the yield of reaction increased four times [180]. Further examples of photo(electro)catalyst type-II heterojunctions are: WO_3_/Cu_2_O [181], In_2_O_3_/CuO [182,183], NiO/Cu_2_O [184], CeO_2_/CuO [185], CdS/Cu_2_O [186], ZnS/Cu_2_O [187] and CuS/CuO [188].

**Figure 15:**
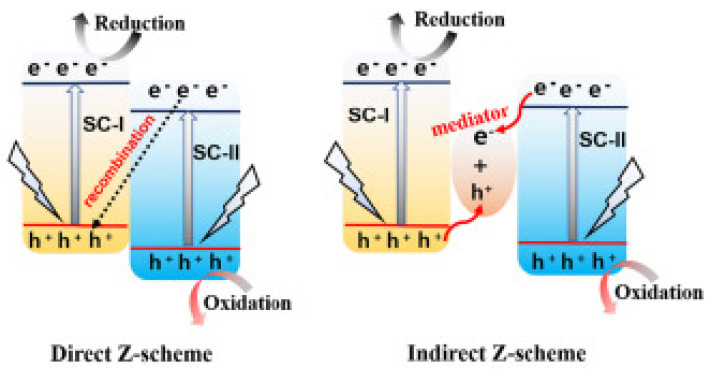
Direct and indirect types of Z-scheme composites. Adopted from [195] with the permission of Elsevier.

Z-scheme is a kind of heterojunction construction proposed to optimize the drawbacks of lower redox potentials of heterojunction system. Specifically, there are two types of Z-scheme composites—direct and indirect (Figure 15). Indirect systems required the presence of an electron mediator to provide an efficient electron transfer between the two semiconductors. Recent literature shows a few examples of Z-scheme composites based on copper oxides. The CuO-Bi-BiOBr system was studied for enhanced sunlight driven alcohol oxidation [189]. In this case, bismuth particles act as an electron mediator and connector between the BiOBr and CuO. Titanium dioxide and copper(I) oxide, with silver nanoparticles as a mediator, form the Z-scheme photocatalyst of water splitting with hydrogen evolution [190]. This system can effectively work also without electron mediator. Titanium dioxide and copper oxide can be connected also using other, more complex compounds (e.g., Ti_3_C_2_Tx [191]). Double-shelled hollow Z-scheme system Cu_2_O@CuCo_2_O_4_ was reported as a photocatalyst for organics degradation [192]. In an interesting approach, a copper-based Z-scheme uses a copper electron mediator to connect copper oxide with other semiconductors. This approach is represented by Cu_2_O/Cu/AgBr/Ag or Cu_2_O/Cu/g-C_3_N_4_, which were tested towards pollutants degradation [193,194]. The latter system is an example of carbon/copper composites, discussed below in detail.

### 2.6. Carbon Based Composites

Because of the narrow band gap, copper oxide nanoparticles are characterized by a relatively high probability of electron-holes pair recombination, which leads to significantly decreased photoactivity. To compensate this drawback, various carbonaceous materials such as carbon nanotubes (CNT), graphene, graphene oxide (GO) or graphitic carbon nitride (g-C_3_N_4_) have gained attention. Carbonaceous materials act in a several ways: They decrease the rate of unfavorable recombination thanks to a mutual charge transition, offer support for anchoring metal oxides and increase the specific surface area. Moreover, generated charge carriers in copper oxides grains cannot be efficiently transferred to the surface due to poor conductivity, so it is meaningful to combine copper oxides with high conductivity materials such as carbon nanotubes [196]. Another advantage of carbonaceous materials is their high chemical stability.

A CuO/CNT composite was prepared by electrolysis and has been tested for the photocatalytic degradation of p-chloroaniline [197]. TEM analyses clearly indicate the tube-shaped materials (CNT) organized in bigger linear structures and the CuO nanoparticles with the crystallite size less than 20 nm. From the FTIR results, bands corresponding Cu–O–C bonds confirmed the interaction between CuO and CNT. Finally, it has been demonstrated that the photoefficiency of p-chloroaniline degradation increases with increasing load of CNT, up to 50 wt%. Other carbonaceous materials like the CNTs/Cu_2_O-CuO photocatalyst was synthetized by a spray pyrolysis method, using copper acetate and CNT dispersion as a substrates [196]. Experimental research demonstrated the numerous effects related to the formation of composite with CNT, such as 30% higher specific surface area and a doubled reaction yield towards degradation of organic dye.

Graphene, graphene oxide and reduced graphene oxide attracted large interest as a support for many photocatalyst, including CuO and Cu_2_O. Such materials have been summarized in numerous review articles [173,198,199]. First of all, combination of copper oxide materials with a highly conductive graphene framework is an attractive approach to assemble efficient photocathodes for solar fuel generation. Kecsenovity et al. described the multiple potential-step electrochemical deposition of copper(I) oxide on the surface of graphene and graphene foams [200]. The PEC activity towards CO_2_ reduction was deeply studied: Graphene-containing photocathodes showed improved properties in comparison with pure Cu_2_O, both in terms of the achieved current densities and stability. Electrochemical analyses demonstrated that the main contribution of the graphene component was the facilitation of charge separation and transport, leading to an improved harvesting of the generated charge carriers. The proposed mechanism of activity includes: (1) Photoinduced electron transition from Cu_2_O to CO_2_; (2) recombination of electrons and holes in the space charge area; (3) hole transfer from copper oxide to graphene, and (4) hole transport to the current collector. Furthermore, graphene-type materials/semiconductor composites were demonstrated also to possess improved optical properties: A notable lowering of band gap energy of Cu_2_O/GO composite with an increasing graphene oxide amount has been observed which is due to the formation of sub-bands between the valence and conduction bands of Cu_2_O, as confirmed by Urbach energy analysis [201].

Among all graphene materials, reduced graphene oxide (rGO) has been attracting the highest attention. rGO is a form of graphene oxide treated by chemical, thermal, photochemical and other methods in order to reduce the oxygen content. Graphene has a serious agglomeration because of the strong interaction between the layers. In turn, graphene oxide has a lowered electrical conductivity due to the oxygen-containing functional groups on the surface. Therefore, thanks to the elimination of oxygen groups, rGO seems to be free of these negative effects. The presence of Cu_2_O nanograins on rGO sheets can successfully ameliorate the agglomeration issue between layers while, thanks to the improved electrical conductivity, photogenerated electrons from the CB of copper oxide can be transferred instantly via rGO sheets leading to a decrease of the unfavorable recombination of carriers [202]. Such promising effects resulted in numerous example of photocatalytic and photoelectrochemical application of copper oxide/rGO: Cu_2_O/rGO and CuO/rGO as visible-light active catalysts of CO_2_ photoreduction to methanol [203,204], Cu_2_O/rGO towards photocatalytic degradation of pollutants [205,206,207] and Cu_2_O/RGO and CuO/RGO nanocomposites for photocatalytic and photoelectrochemical H_2_ evolution [198,199,208].

More complex, multicomponent materials based on copper oxide and graphene oxide were also investigated, for example, Jang et al. reported delafossite CuFeO_2_/NiFe/reduced graphene oxide photoelectrocatalyst with high photoactivity of −2.4 mA cm^−2^ at 0.4 V RHE in NaOH electrolyte [149]. The Cu_2_O/reduced graphene oxide/TiO_2_ (Cu_2_O/rGO/TiO_2_) photocatalyst synthesized under ultrasonic irradiation was studied in “click” reaction for the synthesis of 1,2,3-triazoles via one pot multicomponent reaction of benzyl halide or epoxide derivatives with alkynes under visible light irradiation [209].

**Figure 16:**
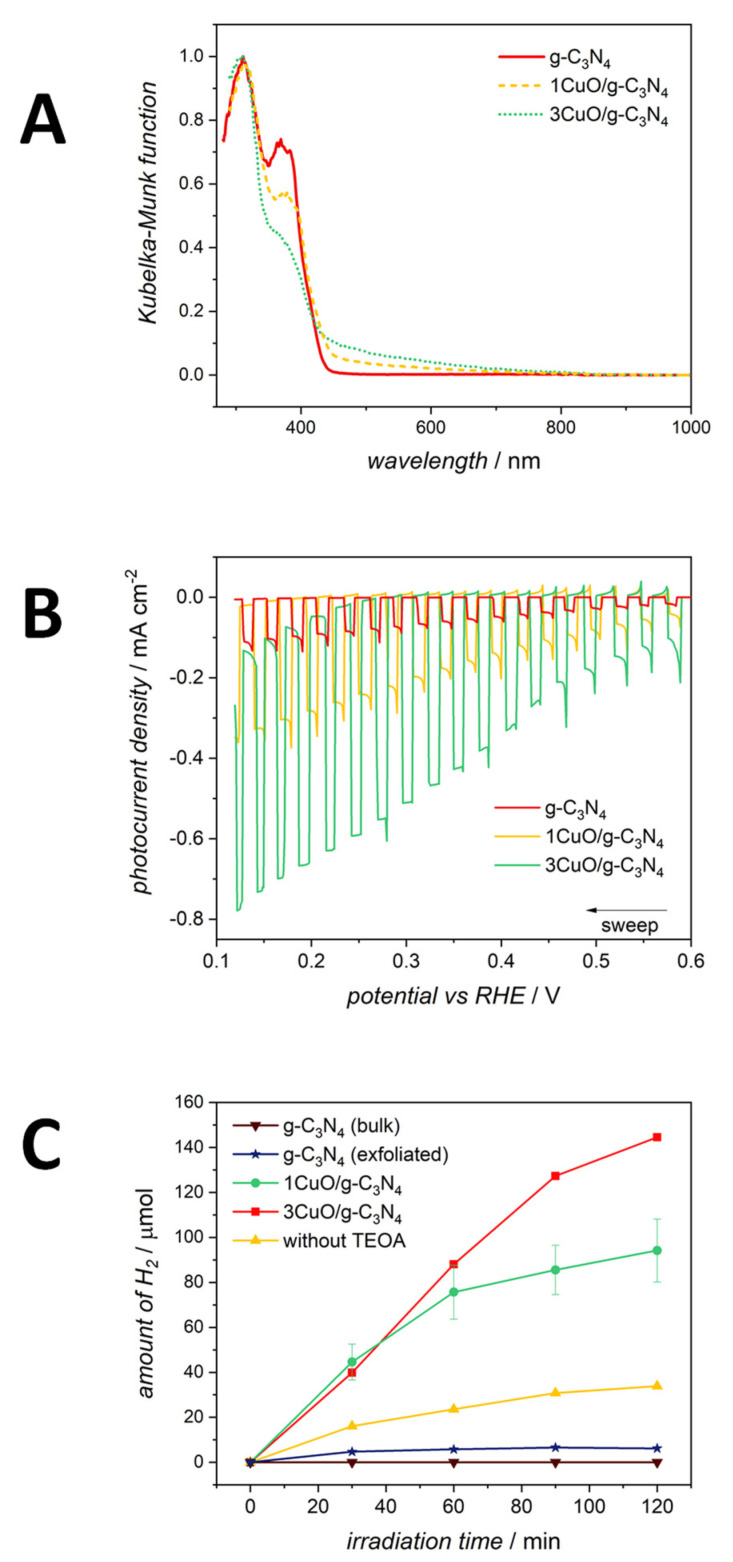
Spectroscopic (**A**) and electrochemical (**B**) study of CuO/g-C_3_N_4_, as well as their photocatalytic activity towards hydrogen evolution (**C**). Reprinted from [210].

Finally, composites of copper oxide with g-C_3_N_4_ were recently reported by Wojtyła et al. [205,210]. CuO/g-C_3_N_4_ photocatalysts have been successfully synthesized by exfoliation of a bulk graphitic carbon nitride and subsequent hydrothermal decoration with CuO nanoparticles. Such methodology resulted in composites with a low amount (up to 5%) of copper oxide at monolayer g-C_3_N_4_. The addition of CuO significantly enhanced the light adsorption in the visible-light region (Figure 16A) and reduces (towards more negative values) the flat band potential. Composites were able to generate high density cathodic photocurrent (Figure 16B), while irradiating the material suspension leads to water splitting with hydrogen evolution even without a sacrificial electron donor (Figure 16C). The improved photocatalytic and photoelectrochemical activity of CuO/g-C_3_N_4_ results from the formation of type-II heterostructure and mutual charge transition [205]. In such heterostructures, the differences of chemical potential between semiconductors result in band bending at the interface of junction, which drives the electrons and holes to move in opposite directions, resulting in a spatial separation of the photogenerated charge carriers on different sides of heterojunction.

In contrast, Mitra et al. and Liu et al. investigated copper(I) oxide/graphitic carbon nitride composites [211,212]. The theoretical calculations demonstrated that the sp^2^ hybridized nitrogen atoms of g-C_3_N_4_ may act as binding sites to connect the Cu_2_O. Spectroscopic studies confirmed the lowering of band gap energy of materials with increasing of Cu content. These nanomaterials were investigated as photocatalysts for organics degradation. Interestingly, it was observed by XPS analysis that the oxidation state of Cu varies with its concentration in the composites [211]. Graphitic carbon nitride and copper oxide can form a Z-scheme system photocatalyst [194].

### 2.7. Surface Modified/Functionalized Copper Oxides

Surface modification of a semiconductor can be performed using organic or inorganic chromophores. Different mechanisms of interaction can be distinguished [206,207]:

- Direct photosensitization (optical charge transfer), observed in the presence of surface metal to metal charge transfer (MMCT) or ligand to metal charge transfer (LMCT);
- An electron injection from the excited photosensitizer to the semiconductor’s CB.
- A hole injection from the excited photosensitizer to the semiconductor’s VB.

The first mechanism involves a direct optical electron injection (OET—optical electron transfer) through the excitation of a photosensitizer-semiconductor complex created upon chemisorption of organic or inorganic ligands onto semiconductor. A LMCT or MMCT are at the basis of the electron injection to the conduction band. The other two mechanisms involve the excitation of the surface bound chromophores followed by an interfacial electron transfer from the excited state of the modifier to the CB of the semiconductor (photoinduced electron transfer) or a hole injection from the excited state of the modifier to the valence band of the semiconductor (photoinduced hole transfer). In both cases, the semiconductor can be in the fundamental or in excited states. Good modifiers should have a strong light absorption, have long lifetimes of the excited states, exist in stable oxidized and reduced forms and do not have the tendency to aggregate or degrade. Regardless of the mechanism, surface modifiers can play various roles (e.g., enlarge the light absorption range, act as an electron mediator or cocatalyst and improve the adsorption of reagents).

The Grätzel group reported Cu_2_O photocathode modified with a rhenium bipyridyl catalyst [213]. Such a system was used towards photoelectrochemical reduction of CO_2_ to CO. It has been found that, upon irradiation, electrons from the CB of Cu_2_O can be transferred through the CB of TiO_2_ (protective layer) to the rhenium complex (Re(tBu-bipy)(CO)_3_Cl), which is a real catalyst of carbon dioxide reduction.

**Figure 17:**
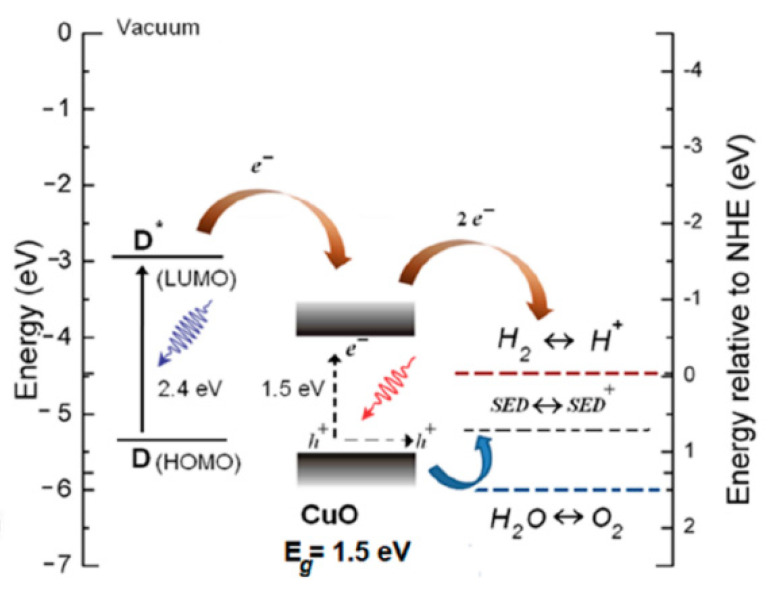
Proposed schemes of light absorption and electron transfer in dye-sensitized CuO. (D*) represents the photoexcited mercurochrome that injects electrons into the CB of CuO thus transforming into the dye cation *D^+^*. Adopted from [214] with permission of the American Chemical Society. LUMO: Lowest Unoccupied Molecular Orbital of mercurochrome; HOMO; Highest Occupied Molecular Orbital of mercurochrome; SED: possible oxidizable chemical specie in solution.

Dye-sensitized copper oxide is the next type of surface modification. Marathey et al. reported mercurochrome-sensitized CuO obtained directly on Cu, which was studied as photocathode in photoelectrochemical cell [214]. Mercurochrome (called also merbromin) is a xanthene dye, an organometallic compound containing mercury atom. Modified nanomaterial exhibits better PEC efficiency (more than two times higher photocurrent density) through an effect of carrier injection from the excited dye to the excited semiconductor (Figure 17). The main advantages of the proposed system are a strong light harvesting ability of the dye and fast electron injection kinetics.

Increased photocurrents were observed also in the case of dye-modified Cu_2_O. A series of dyes have been investigated as electron donors (ZnP-A, C343, N3) or acceptors (C60M-A, NcQ-A, PTCDA) [215]. Experimental results demonstrate that the Cu_2_O/dye/electrolyte interface shows significant differences in the balance of forward/back electron transport in comparison with the pure Cu_2_O/electrolyte interface. In principle, each dye in its basic state should be able of accepting a photogenerated electrons from the conduction band of Cu_2_O (based on redox properties). However, various dyes showed different mechanisms for increasing photocurrents.

Recently, semiconductors modified with MOF (metal-organic framework) structures gained attention. Cu_3_(btc)_2_ (where btc—1,3,5-benzene tricarboxylate)-decorated cuprous oxide nanowires can be given as an example of such system investigated in photoreduction of CO_2_ [216]. The MOF compound leads to the suppression of the corrosion of Cu_2_O and also facilitate charge separation and improves CO_2_ adsorption. Experimental photoluminescence results proved the direct transfer of photogenerated electrons from the CB of Cu_2_O to the LUMO level of non-excited Cu_3_(BTC)_2_. Similar mechanism of improved photoactivity was observed also in case of a more complex system: TiO_2_/Cu_2_O/ Cu_3_(btc)_2_ [217].

## 3. Further Approaches of Photo-Efficiency Improving

### 3.1. Underlayers and Overlayers for Efficient Charge Transport

Cu_2_O is an outstanding p-type photocathode for PEC water splitting since it has a direct band gap of 2 eV and a corresponding theoretical photocurrent of −14.7 mAcm^−2^, and a light-to-hydrogen conversion efficiency of 18% based on the AM1.5 spectrum [218]. However, the redox potential for reduction and oxidation of monovalent copper oxide is within its band gap and the reactions are like Equations (5) and (6), which lead to poor stability and limits its application.

(5) $${\mathrm{Cu}}_{2}{O+H}_{2}O+2e^{-}\;↔{2\mathrm{Cu}+2\mathrm{OH}}^{-}(+0.47\;V\;\mathrm{vs}.\;\mathrm{SHE},\;\mathrm{pH}=0)$$

(6) $${2\mathrm{CuO}+H}_{2}{O+2e}^{-}\;↔{\mathrm{Cu}}_{2}{O+2\mathrm{OH}}^{-}(+0.6\;V\;\mathrm{vs}.\;\mathrm{SHE},\;\mathrm{pH}=0)$$

To inhibit this process, the deposition of a suitable overlayer is the most common strategy to block these reactions, by inhibiting photo-generated electron transfers to the surface of Cu_2_O (and CuO) through insulating the latter from the electrolyte. As a protective layer to passivate the surface of Cu_2_O, the design should follow some guidelines: (1) The layer should have small resistance and the photo-generated electrons and holes can transfer from the Cu_2_O to the solution; (2) this corresponds to a staggered type-II, and then the photogenerated electrons can flow from the Cu_2_O through the protective layer to the electrolyte to promote water reduction, whereas holes flow into the Cu_2_O bulk [48]; (3) the layer should be chemically stable in the reaction conditions. According to these principles, the protective overlayer/underlayer usually forms a n-p heterojunction to allow the transfer of photo-generated holes.

**Figure 18:**
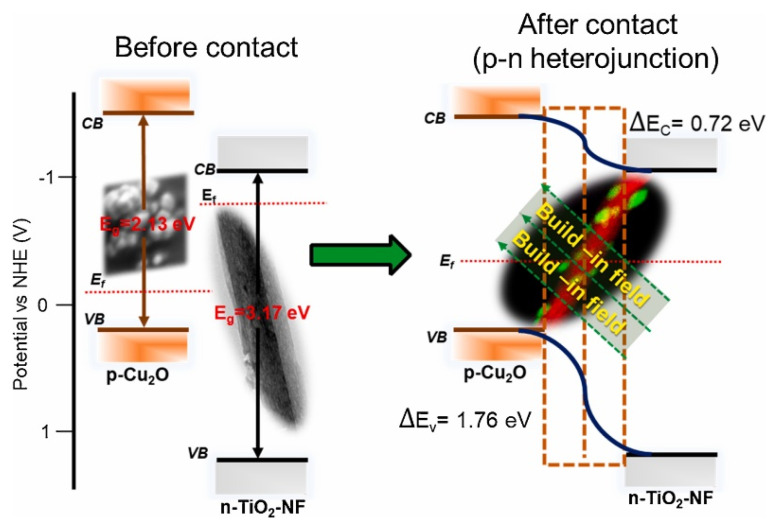
Band alignment energy diagram of Cu_2_O, TiO_2_-NF before and after contact. (CB: Conduction band, VB: Valence band, *E*_f_: Fermi level, Δ*E_C_*: Conduction bands offset, Δ*E_V_*: Valence bands offset). Reprinted from [229] with the permission of Elsevier.

After illumination, electron-hole pairs are generated, and the photo-generated electrons will transfer from the conduction band of the p-type Cu_2_O to the n-type semiconductor, where they accumulate in the conduction band. This extends the lifetime of electrons, thus inhibiting charge recombination. Besides, photogenerated holes will transfer from the n-type semiconductor to Cu_2_O. As a result, this mechanism overcomes charge recombination, and then the photo-generated electrons have more chance to be involved in the desired reduction process. Quite importantly, the valence band of p-type Cu_2_O is just slightly lower than the potential of oxygen evolution. However, after being in contact, the valence band of the n-type semiconductor can better meet the potential for oxygen evolution reaction (OER) [219]. Thence, the p-n heterojunction can enhance the performance and block the self-oxidation of Cu_2_O as a protective layer. Many n-type semiconductors, Ga_2_O_3_ [220,221,222,223], ZnO [224,225], TiO_2_ [226,227] and NiO_x_ [50], deposited over Cu_2_O can enhance the performance, since the n-type semiconductor and p-type semiconductor can provide holes and electrons for the water splitting, respectively, as well as relative band positions. Several Cu_2_O-based n-p heterojunctions behave as schematized in Figure 18. After the contact, a built-in electric field is set, which can lead the photo-generated electrons transport thus prolonging the electrons lifetime and improving charge separation. In addition, this combination will enhance the light adsorption edge. Paracchino et al. adopted atomic layer deposition (ALD) to deposit TiO_2_ as a protective layer on Cu_2_O and forming a highly active photocathode for solar H_2_ production, which can protect against photocathodic decomposition. As a result, 5 *(4 nm ZnO/0.17 nm Al_2_O_3_)/11 nm TiO_2_ (it stands for five bilayers of 4 nm ZnO and 0.17 nm Al_2_O_3_, followed by 11 nm TiO_2_) showed best photocurrents of up to −7.6 mA cm^−2^ at a potential of 0 V vs RHE, and it can also remain active after 1 h test under AM 1.5 illumination at neutral pH. Meanwhile, the Faradaic efficiency was close to 100% [48]. Zhang et al. use a covalent triazine frameworks (CTF-BTh) containing a bithiophene moiety as a protective layer to deposit onto the surfaces of a Cu_2_O photocathode. At the same time, they constructed a Mo-doped BiVO_4_ photoanode via electropolymerization. CTF-BTh possesses a suitable band structure for a p–n junction and a staggered type-II heterojunction with Cu_2_O and Mo-doped BiVO_4_, respectively, which is a benefit to the water splitting process. Lastly, the CTF-BTh film plays an important role as an effective corrosion-resistant overlayer for both photoelectrodes to inhibit photocorrosion, and it lasts for long-term operation for 150 h with only ≈10% loss in photocurrent densities. Surprisingly, a standalone unbiased PEC tandem device comprising CTF-BTh-coated photoelectrodes exhibits 3.70% solar-to-hydrogen conversion efficiency and keeps continuous operation for 120 h, the efficiency can still retain at 3.24% [228].

A second strategy to protect the semiconductor is to deposit a hole transfer layer as an underlayer (i.e., between the conductive glass support and Cu_2_O). In this way, holes will transfer from Cu_2_O to the hole transfer layer to inhibit the recombination of photo-generated electron and hole pairs, thus overcoming the self-oxidation of Cu_2_O and enhance the performance of PEC water splitting. Pan et al. designed a Cu_2_O-based photocathode, using Ga_2_O_3_ and Cu_2_O forming a n-p heterojunction, as well as two different structures with CuSCN as the hole transport layers buried with TiO_2_ as a protective layer. Since the conduction band edge of CuSCN is at a higher potential than that of Cu_2_O, with a 2 eV band gap difference, a huge barrier for electrons transfer from Cu_2_O to CuSCN (towards the counter electrode) is generated. Enlarged XPS valence band spectra illustrated that an acceptor-like state near the Fermi level is generated and this can enhance the hole conductivity. Although CuSCN and Cu_2_O have very close valence band edges, the existence of band-tail states leads to a smooth hole transport, thus decreasing the probability of electron-hole recombination. As a result, this combination provides a solar-to-hydrogen efficiency of 4.55%, which is the highest efficiency among Cu_2_O based photocathodes [221]. Besides, other semiconductors can also be used as hole transfer layer, such as FeOOH [230] and NiO [231]. Zhou at al. firstly explored mechanism behind the stability of Cu_2_O and demonstrated that the accumulation of photogenerated holes in the valence band of Cu_2_O is a key factor. In this work, FeOOH was adopted as a hole transfer layer (HTL) and Pt was added as a cocatalytic layer (see below). As a result, photo-generated holes are transferred from the VB of Cu_2_O to FeOOH and then to a Pt counter-electrode through the external circuit. At the same time, photogenerated electrons are transferred from the conduction band of Cu_2_O to the Pt cocatalytic layer. The synergistic effect of the Pt layer and of FeOOH inhibits the recombination of photogenerated electrons and holes, which not only retard the self-oxidation of Cu_2_O, but also promotes the photoelectrocatalytic activity of Cu_2_O [230].

In addition, metals can be used as underlayers to improve Cu_2_O performances, and the most adopted ones are certainly Au [221] and Cu [53]. Cu_2_O-based photocathodes still employ Au as back contact due to the matching between gold work function and Cu_2_O valence band energy level. Low-cost Cu can replace Au, Cu/Cu_2_O showing similar performances to Au/Cu_2_O [53].

In summary, constructing a protective layer to overcome the photocorrosion of Cu_2_O and improve its performance mainly depends on enlarging the light adsorption edge, improving the separation efficiency of electron-hole pairs, changing the electron transfer path as well as prolonging the electrons’ lifetime.

### 3.2. Deposited Cocatalyst (Pt, Ag)

A transition metals, in particular noble metals are loaded on the semiconductors as a cocatalyst to enhance the photoefficiency of reaction. Cocatalysts deposited on surface of semiconductor can provide reaction active sites, catalyze the surface reactions by lowering the activation energy, trap the charge carriers (electron sink role) and suppress the unfavorable recombination of charges [232].

**Figure 19:**
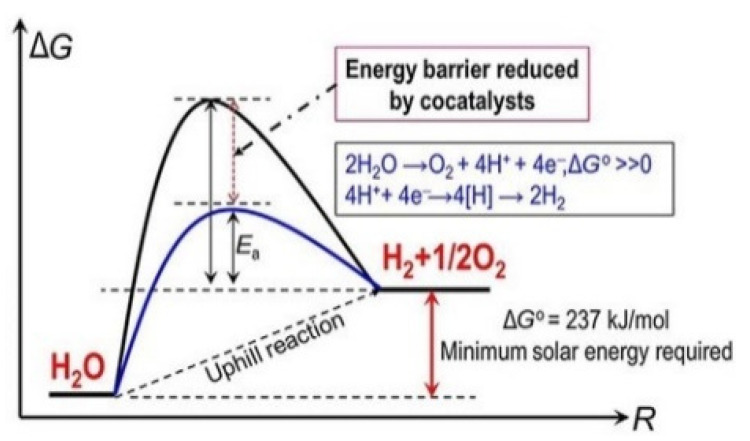
Schematic description of the functions of cocatalysts from [232], with permission of the American Chemical Society.

Cocatalyst may offer an alternative reaction mechanism characterized by a lower activation energy in comparison with the non-cocatalyzed pathway. For example, the theoretical minimum Gibbs free energy required for a water-splitting reaction is 237 kJ/mol. In practice, significantly higher energy is necessary to overcome the kinetic barriers for both HER and OER on photocatalyst. Proper cocatalysts can successfully reduce such energy barriers by lowering the activation energy (Figure 19). Particularly, water reduction usually requires much lower overpotential than water oxidation, so the role of cocatalyst in OER processes is more crucial. A direct correlation between the PEC yield and properties of the cocatalysts has been reported [233]. Among noble metals, platinum with higher redox potential and work function was identified to be the most favorable for HER. Instead, a volcano relationship between the exchange current for HER and the metal-hydrogen bond energy was proposed by Trasatti, and platinum showed the lowest activation energy for HER [234]. Therefore, Pt is considered as the most suitable cocatalyst for PEC H_2_ evolution from the viewpoint of electronic and catalytic properties.

In regard to improved charge separation effects when a cocatalyst is loaded on the semiconductor, a depletion layer at the cocatalyst/semiconductor interface is formed. A built-in electric field caused by the depletion layer can result in a driving force for efficient separation of the electron-hole pairs. That is to say, a suited cocatalyst enhances the efficiency of photogenerated charge separation and leads to a lowered rate of unfavorable recombination, a major reason for low photocatalytic activity.

Cocatalysts can be deposited on the surface of semiconducting materials using various methods: electrodeposition, photodeposition, wet impregnation, chemical reduction (e.g., impregnation-reduction method), atomic layer deposition, chemical vapor deposition, sputtering, glancing angle deposition, lithography and others. It has been proved, that the method of cocatalyst deposition may affect its role and effectiveness [235].

Recent literature offers numerous examples of cocatalyst deposited on copper oxide materials, both Cu_2_O and CuO. The cocatalysts can be classified into two roles: the cocatalyst trapping electrons for reduction half reactions (e.g., reduction of H_2_O or CO_2_) and the cocatalyst trapping holes for oxidation half reactions (e.g., oxidation of H_2_O or organic species). In general, noble metals (e.g., Pt, Pd, Rh and Au, Ru) [39,48,236,237,238,239,240], non-noble transition metals (e.g., Cu, Zn, Al, Co and Ni) [184,241,242,243,244,245], metal oxides (RuO_2_, NiO_x,_ Bi_2_O_3_) [80,190,246,247,248] and metal sulfides (e.g., ZnS, MoS_2_, NiS, Ag_2_S, and WS_2_) [187,245,249,250,251] were reported as cocatalysts deposited on CuO and/or Cu_2_O.

Deposition of Pt or other cocatalysts on the surface of copper oxides resulted in a few effects. The most basic effects can be observed using photocurrents analysis. Copper oxide with deposited platinum showed significantly higher, even twice higher photocurrents densities [7,25]. Moreover, the photocurrent onset is shifted towards more positive potentials [240]. Xing et al. studied the influence of platinum on the photocorrosion of copper oxide [252]. They proved that Pt hinders the self-reduction of copper(II) oxide because of its fast electrons trap ability. Impedance studies on CuO/Pt photocathodes indicate that the photoinduced electrons transfer from the semiconductor to the electrolyte occurs more easily due to the presence of platinum, thereby suppressing the photocorrosion of CuO.

### 3.3. Surface Plasmon Enhancement Effects

**Figure 20:**
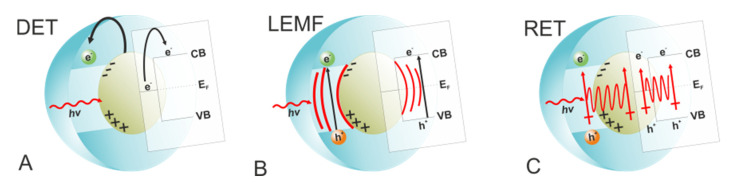
Various mechanism of plasmon energy transfer from metal to the semiconductor. (**A**) Direct electron transfer (DET) of hot electrons, (**B**) local electromagnetic field enhancement (LEMF) of the semiconductor charge separation process, (**C**) resonant energy transfer (RET).

In parallel to cocatalytic effect, the surface plasmon resonance can offer a new opportunity to overcome the low efficiency of photocatalytic reactions [253,254]. Light excitation of a metallic nanostructures leads to a strong optical near-field that induces a cascade of processes with multiple outcomes, including the excitation of surface plasmons, their radiative decay to photons, and their nonradiative decay in the material. The presence of plasmonic metal nanostructures on the surface of semiconductor photocatalysts can improve the photocatalytic efficiency due to: (i) Extending light absorption to visible light due to local dielectric environment alterations, (ii) increasing light scattering (metallic particles scatter incident light and locally amplify the electromagnetic field when placed on the surface of semiconductor), and (iii) exciting electron−hole pairs in the semiconductor by transferring the plasmonic energy from the metal to the semiconductor with various proposed models of energy transfer, as shown in Figure 20. Direct electron transfer (DET) from the metal to the semiconductor’s CB may occur only if they are in direct contact and DET depends on the mutual alignment of the CB and Fermi level of the metal. Another suggested mechanism of energy transfer from plasmonic metal is called surface plasmon resonance mediated local electromagnetic field LEMF and can lead to charges generation in the semiconductor. The charge separation induced by LEMF may form carriers only for energies above the band gap of the semiconducting material. LEMF increases the rate of interband transitions in the semiconductor because of the rise of local electromagnetic field. Moreover, resonant energy transfer (RET) was suggested in the literature as an alternative, nonradiative mechanism of surface plasmon resonance induced charge separation in semiconductors, in contrast to the radiative LEMF model [253]. This process directly induces the electron and hole in the semiconductor nonradiatively by the relaxation of the localized surface plasmon dipole. Described proposed mechanism are not clear and they require additional study in order to find their influence on photocatalytic reaction efficiency.

Surface plasmon effects are typically observed for metals such as gold or silver. The CuO-Au nanohybrids showed enhanced visible light absorption properties, four times higher photocurrents and significantly improved kinetic of reaction [246]. The enhanced photocatalytic activity was attributed to the surface plasmon resonance effect of Au NPs. Similar, thin films, composed of bimetallic Au-Ag nanoparticles deposited on a CuO showed Localized Surface Plasmon Resonance (LSPR) responsible for significantly improved light absorption [255]. Hajimammadov and co-workers proved surface plasmon effects in case of copper metallic nanoparticles on Cu_2_O/CuO composite [256].

## 4. Application of Copper Oxide-Based Materials in Photocatalysis and Photoelectrocatalysis

### 4.1. H_2_ Evolution

In the future of hydrogen economy, photoelectrochemical water splitting is one of the most promising alternatives for renewable hydrogen generation and storage. The process simply needs water, sun and a semiconducting material. Obviously, the photocathode and photoanode must have the appropriate band gap and bands alignment with respect to the HER and the OER, respectively. In the working configuration, the semiconductor was immersed in a water-based electrolyte, with sunlight being the driving force for charges generation inside valence and conduction bands. These charges are the energy source for the water splitting reaction.

Under light, photons possessing more energy than *E*_g_ are absorbed by the material and electrons are excited from the VB to the CB, generating electrons-holes couples. An applied electric field can force the separation between photogenerated electrons and holes. In doped semiconductors, band banding is generated after the immersion in the electrolyte and induced a potential gradient within the material. In a p-type material, this attracts electrons towards the solution, while holes move toward the bulk semiconductor and then to the counter electrode. The use of an external bias can be useful to overcome eventual overpotentials, to improves the band bending giving an efficient charges separation or to compensate for the insufficient position of band edges with respect to the semi-reactions of interest [257,258]. There are some requirements for a PEC-WS system to work properly:

- An efficient absorption of solar light to produce excited states in the semiconductor.
- A good charge separation to avoid recombination of the electron-hole couple and ensure a high light-to-chemicals conversion.
- Proper bands position with respect to HER and OER from thermodynamic and kinetic points of view.
- High stability to photodegradation processes.

Accomplishing all of these needs is not simple. Many semiconductors able to perform the desired reaction without undergoing photodegradation show a wide band gap that reduces the portion of solar spectra available for the absorption (e.g., TiO_2_ with a 3 eV band gap can absorb only in the UV) [7]. Despite this, compared to other H_2_ production routes, PEC-WS has the advantage of having a relatively simple and cheap process. It does not require expensive setups, is completely based on renewable sources and has no by-products. It can also work in wide range of temperatures and without a warm-up step (if the temperature is above 0 °C). Once all the requirements are satisfied at the photoanode, reaction (1) takes place.

While at the photocathode, water reduction will occur using the high-energy electrons excited in the CB by the solar light (Equation (2)).

The so produced hydrogen can be collected and stored to be used when required for examples in fuel cells or as a chemical in the oil industry.

**Figure 21:**
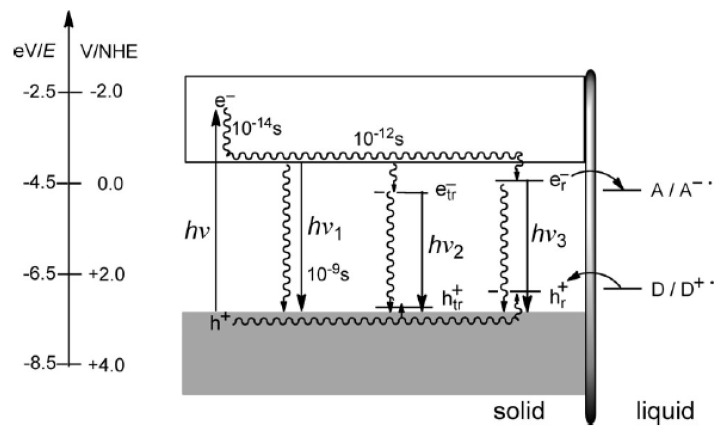
Schematic representation of the possible pathways that electrons and holes can follow after the irradiation of the semiconductor. Reprinted from [259] with permission from John Wiley and Sons.

The desired electrons transfer is not the only available route for holes and electrons. Figure 21 summarizes the possible processes following the photon absorption in a semiconductor immersed in an electrolyte with a donor D and an acceptor A. Once the light-excited electrons and holes are moved at the corresponding edges of energy bands they may undergo trapping (reactive and non-reactive) on surface sites as well as radiative and nonradiative recombination. These alternative routes for the electron-hole couple will reduce the conversion efficiency of sunlight to chemical products.

In materials like Cu_2_O, but also ZnS or CdS, the main path for the material photodegradation (photocorrosion) is the electron-transfer reaction to the material itself (reaction not included in the scheme). This happens when the redox potential of the material lies within the band gap. As a result, electrons (but also holes) react with the semiconductor itself leading to a reduction to the corresponding metals. This photo-degradation reaction is even stronger for very pure materials suggesting the importance of doping [259].

The main PEC-WS challenges that have to be overcome in this system are:

- Extended lifetime for photoelectrode materials and thus limited (photo)corrosion processes.
- The right bands edge alignment for the redox reactions from a thermodynamic point of view.
- Fast kinetics for HER/OER.
- A suitable optical band gap for optimal light absorption and high photon efficiencies.
- A fast charges transport in the bulk semiconductor.
- Low internal electrical resistance.
- Limited phenomenon like trapping and recombination (see Figure 21).
- Low-cost materials with wide availability and reduced plant capital costs.

The semiconductor-based PEC-WS is nowadays an almost mature technology to be a valid solution for near-future energy storage. Despite the high cost for the H_2_ production in PEC-WS systems, the continuous improvements in the field are promising for the future scalability of this technology.

### 4.2. CO_2_ Reduction to Methane and Other C1 Compounds

The use of sunlight to convert carbon dioxide into fuels, such as methanol, methane or formic acid, might contribute to solving two global problems: high emission of CO_2_ into the atmosphere and depletion of fossil fuels [247]. In heterogeneous photocatalytic reduction of CO_2_, the reaction takes place at a solid-gas or solid-liquid interface, and the photocatalysts are often hybrid materials realizing several tasks: (i) Light absorption, (ii) separation of the photogenerated charges, (iii) transfer of charges to the surface and (iv) redox reactions at active sites. The adsorption of carbon dioxide on a photocatalyst surface is the first step of photocatalytic activation of CO_2_, that leads to the formation of a partially charged species (CO_2_^δ•-^) and loss of the linear symmetry of the molecule. Consequently, the barrier for accepting an electron in further photocatalytic process is lowered [248]. The electron transfer from the excited semiconductor to the CO_2_ molecule adsorbed at the material’s surface initiates the cascade of processes involving the cleavage of C-O bonds and formation of C-H bonds. One electron reduction of CO_2_ to its anion radical is a thermodynamically unfavorable reaction, in part because the additional electron must occupy the lowest energy π* orbital of CO_2_ [260]. The addition of one electron causes bending of the molecule from 180° to about 120°. The loss of symmetry is related with a repulsion between free electron pair at the oxygen atoms and one extra electron localized at the carbon atom. Direct one-electron reduction of CO_2_ leads to the formation of the carbon dioxide radical anion CO_2_¯. The redox potential of the CO_2_/CO_2_¯ couple in water amounts to −1.9 V vs. a standard hydrogen electrode and, therefore, only semiconductors characterized by particularly negative potential of conduction band can be used as a photocatalyst of one-electron reduction of CO_2_ (e.g., ZnS or SiC) [260,261,262].

In contrast, a proton-assisted multielectron reduction of carbon dioxide leads to a variety of stable products making such reactions thermodynamically preferred. The number of transferred electrons (up to eight) determines the final oxidation state of the carbon atom [263]:

CO_2_ + 2H^+^ + 2e^−^→ HCOOH E° = −0.250 V (7)

CO_2_ + 2H^+^ + 2e^−^ → CO + H_2_O E° = −0.106 V (8)

CO_2_ + 4H^+^ + 4e^−^→ HCHO + H_2_O E° = −0.07 V (9)

CO_2_ + 6H^+^ + 6e^−^ → CH_3_OH + H_2_O E° = 0.016 V (10)

CO_2_ + 8H^+^ + 8e^−^ → CH_4_ + 2H_2_O E° = 0.169 V (11)

The redox potentials of CO_2_ reduction to CO, HCOOH, HCHO, CH_3_OH or CH_4_ are noticeably higher (less negative) than the conduction band edge potential of many common semiconductors (e.g., CuO, Cu_2_O or TiO_2_), thus photocatalysis can be successfully applied to these processes. These strategies of carbon dioxide reduction can be performed also in a photoelectrochemical way, as discussed in more detail elsewhere [264].

### 4.3. Degradation of Organic Species in Liquid or Gas Phase

Photocatalytic degradation of organic species such as VOC (volatile organic compounds), dyes, pesticides or pharmaceuticals is an approach of environment remediation using a broad variety of photoactive materials. Technology readiness levels of these processes are very high, and many semiconducting materials demonstrated acceptable photocatalytic efficiency in a real space environment. Removal of indoor VOC pollutants has been already implemented in the economy, since photocatalytic air purifier or purification systems are commercially available. Process of organic compounds degradation includes a few stages: (i) Mass transfer of the organics to the surface of the photocatalyst, (ii) adsorption of the molecules onto the active sites, (iii) photocatalytic degradation, (iv) desorption of the products of degradation and (v) mass transfer of these products away from the photocatalyst’s surface [265].

**Figure 22:**
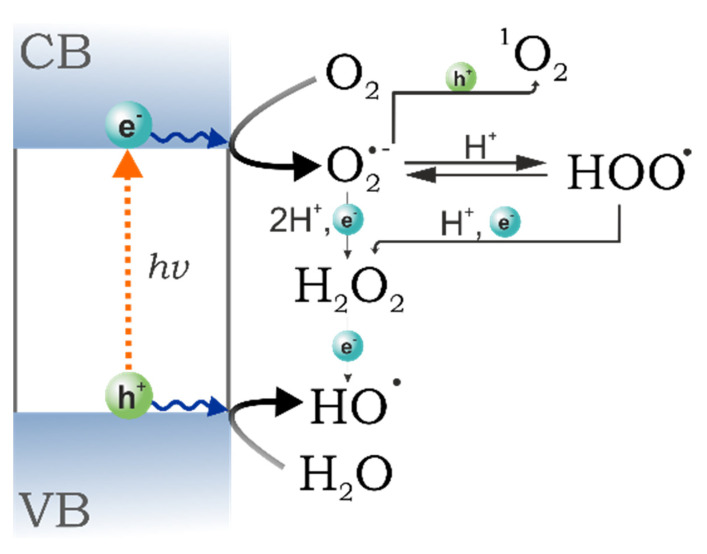
Schematic mechanism of photocatalytic formation of ROS from [268] with permission of American Chemical Society.

The mechanism of photocatalytic degradation of organic species is not simple because it involves two main steps. Semiconductors absorb light to generate pairs of electrons and holes. These charges react with oxygen or water (steam) to form reactive oxygen species (ROS), such as hydroxyl radicals, superoxide anion radicals, singlet oxygen, hydrogen peroxide and others. The mechanism of ROS formation via photocatalysis is well know from the literature and has been shown in Figure 22 [266]. In a second step, ROS are the key oxidants that decompose organic pollutants via the so-called advanced oxidation processes. ROS possess higher reaction activity than O_2_ and can completely destroy a very broad range of organic pollutants including organic halides and polycyclic aromatic hydrocarbons. At the same time, some organics can be directly oxidized by photogenerated holes [267]. Photocatalytic oxidation of VOC and other organic compounds leads to safe products such as CO_2_, CO and H_2_O.

Despite many advantages, photocatalytic purification of air or wastewater has serious limitations, such as a slow rate and low quantum efficiency. More information on the photocatalytic degradation of VOC in gas phase or decomposition of dyes, pesticides or pharmaceuticals in liquid phase can be found in recent reviews [265,268,269,270].

### 4.4. Other Photocatalytic Processes

Copper-based materials can be used as a photocatalyst also in many other photocatalytic processes such as: removal of heavy metals from wastewater, photocatalytic abatement of NO_x_ pollutants in the air or photo(electro)catalytic regeneration of cofactors such as NADH (nicotinamide adenine dinucleotide coenzyme).

Metal ions are generally not decomposed, but various photocatalytic strategies have been suggested to eliminate heavy metals from wastewater, in particular, reductive and oxidative strategies [271]. Heavy metals and metalloids removal from water basically involves reduction process that produce elemental metals (can be easier removed by other techniques) or metal ions at a lower oxidation state, that are less toxic (e.g., toxic chromium(VI) reduced to non-toxic chromium(III) [272]). The oxidative strategy can be considered in case of arsenic, which occurs in the anionic form and has to be oxidized to be removed [271].

Regenerated NAD cofactor (NADH) can be considered as a form of energy storage that can be used, for example, in enzymatic conversion of CO_2_ to methanol. Enzymatic reduction of carbon dioxide is catalyzed by some enzymes, such as formate dehydrogenase, formaldehyde dehydrogenase and alcohol dehydrogenase, while the necessary energy is provided by NADH [273]. In each enzymatic step, one mole of NADH is oxidized to NAD^+^ that must be converted back to NADH. This regeneration can be performed in a photoelectrochemical or photocatalytic way, in a simple system or with an electron mediators such as a ruthenium complex [273,274].

## 5. Conclusions

In this review we analyzed and discussed the use of Cu-based materials as photoactive materials in different reactions, including water splitting, CO_2_ reduction and the remediation of wastewaters. Cu is a relatively abundant element and its compounds can be easily prepared and manipulated. Playing on the oxidation state of Cu and the possibility of synthetizing ternary and quaternary oxides, as well as of tuning the electronic properties of these materials by doping, it is possible to prepare a semiconductor with the desired characteristics.

Finally, the performances of the final material can be further improved by addition of an overlayer or underlayer, of a cocatalyst or by a combination with a carbonaceous material. We are still far from a material that can sustain the needs of an industrial-level photochemical or photoelectrochemical device, but we here demonstrate that researchers have jumped big leaps in the last five to 10 years by proposing highly active and stable materials, and by better understanding the principle that we should follow for synthetizing the new generation ones.

We can conclude this review in saying that, thanks to these findings, it is now possible to optimize the design of a Cu-based photocathode by acting on the synthesis (with a preference for low-cost, easy scalable methods), doping and addition of under/overlayers and cocatalysts/plasmonic effect-inducing particles. This, in turn, will not be an easy task, mostly due to the increasing number of variables, such as the nature of the precursor, the semiconductor synthesis method and its parameters, the synthesis method and its parameters for the underlayer, the synthesis method and its parameters for the overlayer, the synthesis method and its parameters for the cocatalyst and, finally, the possible methods to build this complex architecture. We foresee that the adoption of combinatorial chemistry methods aimed at the rapid screening of material libraries’ activity [275,276,277] could represent a great help in this sense.

## Figures and Tables

**Table 2:** General physicochemical properties of Cu_2_O. From the Safety Data Sheet of Cu_2_O.

Parameters	Values	Units
Density	6.10	g·cm^−3^
Molar mass	143.092	g·mol^−1^
Molar volume	23.46	cm^3^·mol^−1^
Appearance	Reddish-brown	
Solubility (in water)	insoluble	
Melting point	1232	°C
Boiling point	1800	°C
Band gap	2.17	eV
R-phrases	R22, R50/53	
S-phrases	(S2), S22, S60, S61	
Thermal conductivity [29]	4.5	W·K^−1^·m^−1^
Specific heat capacity [29]	70	JK^−1^·mol^−1^
Thermal diffusivity [29]	0.015	cm^2^·s^−1^

**Table 3:** Cu vacancy formation energy and effective hole masses for configuration with 2 Cu vacancies. Data from [90], with the permission of Elsevier.

Configuration	Electronic State	E^vac^/eV Per Cu	E_g_/eV	m*/m_e_
Clustered same network	Triplet	0.66	0.58	−1.44, −1.38
Clustered different network	Triplet	0.24	0.62	−1.26, −18.20
Clustered same network	Singlet	0.62	0.59	−1.6, −0.51
Clustered different network	Singlet	0.38	0.57	−1.34, −4.55
Isolated same network	Triplet	0.43	0.65	−0.45, −1.15
Isolated different network	Triplet	0.42	0.58	−0.45, −0.45
Isolated same network	Singlet	0.38	0.62	−0.54, −0.50
Isolated different network	Singlet	0.37	0.58	−0.46, −0.45

**Table 4:** Ionic radius and Cu vacancy formation energy (E^vac^) and band gaps (E_g_) for cation-doped Cu_2_O from [31], with permission from the American Chemical Society.

Dopant	Ionic Radius (Å)	Experimental E_g_ (eV)	E_n−1_^vac^ (eV)	E_n_^vac^ (eV)	Computed E_g_ (eV)
Cu_2_O	0.93	2.17	0.36	0.24	0.52
Sn^2+^	0.93	3.05	−0.95	0.15	0.63
Zn^2+^	0.40	3.30	−0.78	0.22	0.49
Mg^2+^	0.49	7.80	−0.46	0.26	0.56
Ca^2+^	1.00	7.03	−0.62	0.23	0.63
Sr^2+^	1.16	5.90	−0.65	0.14	0.65
Ba^2+^	1.36	4.10	−0.66	−0.12	0.62
Cd^2+^	0.84	2.35	−0.28	0.18	0.45
Hg^2+^	0.96	2.20	0.06	0.13	0.30
Al^3+^	0.39	8.80	−0.30	0.36	0.36
Ga^3+^	0.47	4.95	−0.08	0.14	0.11
In^3+^	0.79	3.57	0.16	0.29	0.13
La^3+^	1.06	5.40–5.80	−0.76	−0.57	0.67
Ti^4+^	0.61	3.05	0.12	0.42	0.27
Cr^4+^	0.44	3.25	0.22	0.34	0.04
V^4+^	0.36	0.70	−0.02	0.15	0.04
Ce^4+^	0.80	2.40	−1.26	0.51	0.05

**Table 5:** Electronic properties of copper-based ternary oxides.

Material	Band Gap	Conduction Band Edge	Reference
CuFeO_2_	1.5 eV	−0.4 V vs. RHE	[155]
CuCrO_2_	2.4 or 3.7 eV (depending on defects)		[157]
CuAlO_2_	1.4 eV	−0.24 V vs. RHE	[158]
CuGaO_2_	1.4 eV		[154]
CuRhO_2_	1.9 eV		[159]
CuBi_2_O_4_	1.8 eV	−0.3 V vs. RHE	[160]
